# Subjective social integration and its spatially varying determinants of rural-to-urban migrants among Chinese cities

**DOI:** 10.1038/s41598-024-55129-y

**Published:** 2024-03-06

**Authors:** Qilong Chen, Chengxiang Wang, Pinrong He, Anning Cai

**Affiliations:** 1 Institute of Land and Urban-Rural Planning, Huaiyin Normal University, Huai’an, 223300 China; 2 Urban Design Analysis Lab, Graduate School of Urban Studies, Hanyang University, Seoul, 04763 Korea; 3 School of Tourism and Administration, Nanjing XiaoZhuang University, Nanjing, 211100 China

**Keywords:** Psychology and behaviour, Human behaviour

## Abstract

Social integration, a huge issue triggered by migration, leads to potential social fragmentation and confrontation. Focusing on the precise enhancement of "inner" subjective social integration is the ultimate urbanization solution to enhance people-centered well-being and promote full social integration. This article used data from the China Migrants Dynamic Survey 2017 (CMDS 2017) to reveal the spatial patterns and mechanisms of subjective social integration in Chinese cities. We make an innovative attempt to introduce multiscale geographically weighted regression (MGWR) to address the appropriateness of policy formulation by addressing the spatial variation in the factors. The results demonstrate that the influences on subjective social integration have a strong spatial heterogeneity in China, a vast and unevenly developed country. Expanding on the typical factors, household registration and political participation affect North China more than other regions; and housing and marriage have a greater impact in South China, especially in the Pearl River Delta and the Eastern Seaboard. Income, welfare, and healthcare are indiscriminately sweeping through most of China. Such a conclusion reminds the Chinese government that it needs to consider not only addressing some of the national constraints to subjective social integration but also imposing precise, site-specific changes for different regions.

## Introduction

Social differentiation and social integration, caused by the increasing population mobility, are important issues that are common in the context of global urban transformation^1–3^. The long-term transnational migration since the Industrial Revolution has brought great dynamism to the global North, but it has also bred conflicts between social subjects, i.e., human beings, a case in point being racial tensions. Further globalization and urbanization have created new challenges for the global South. Among these, the vast and hyper-populated country of China also has enormous problems of population social integration, especially facilitated by compressed-explosive urbanization. As of 2021, China’s migrant population had rose to 385 million, according to the China’s Statistical Bulletin of the National Economic and Social Development^4^. As a unique mega-complex, China’s rural-to-urban migrants encounter enormous institutional, economic, cultural, and social obstacles in urban society^5,6^.

In the twenty-first century, with the abolition of the custody and repatriation system (CRS) and the loosening of restrictions on urban settlements, People's migration in China has become "free and unfettered." However, at first glance, the movement of Chinese peasants to the megacities seems more like a "desperate attempt to make a living". As new citizens gradually take root in China's cities, social integration has gradually shifted from passive to active and from objective to subjective. Some of them began to love their new homes and developed a stronger interest in the host city’s development, becoming individuals who no longer differ from the old citizens. However, a considerable portion of the migrant population does not quite recognize their citizenship and integration, often referring to themselves as "peasant workers" and planning to leave at any time. Against the backdrop of the above issues, social fragmentation within cities not only leads to superficial contradictions of social stability, but also has the potential to trigger a deep-seated urbanization collapse or premature counter-urbanization, which is not conducive to people's "sense of well-being".

Factually, subjective social integration usually has implicit characteristics, including psychosocial aspects involving identity, subjective internalization and satisfaction. Consequently as different cities in China possess different developmental status, the degree of social integration of migrant populations varies and the impact of various influencing factors plays different roles^6^. Most of the existing relevant studies focus on the situation of individual residents and families, but objective aspects of cities such as policies and the level of urban economic development, for example, are equally important. Therefore, it should be emphasized that complex regional and individual differences should be taken into account when formulating policies^7^.

On this basis, we utilize data from the China Migrants Dynamic Survey 2017 (CMDS 2017) to examine subjective social integration in 246 Chinese cities. Most importantly, this unique data set is able to categorize subjective integration into three progressive levels of integration WILLINGNESS, LIKING, and CONCERN, which in turn assesses people's feelings from the bottom up. Furthermore, we analyzed the spatial heterogeneity of the factors influencing subjective social integration using the MGWR model, which contributes to the authentic formulation of differential policies.

The remainder of this article is structured as follows. First, Sect. “Literature review” systematically reviews the established literature related to social integration, especially on subjective social integration, and presents our research framework. Next, we introduce the data, the model, and the selected indicators in Sect. “Methodology”. Section “Results” shows the empirical results of this article, which are mainly based on the MGWR test for spatial heterogeneity of the influencing factors. The final Sect. “Discussion and conclusions” comprises the discussion and conclusion.

## Literature review

**Figure 1:**
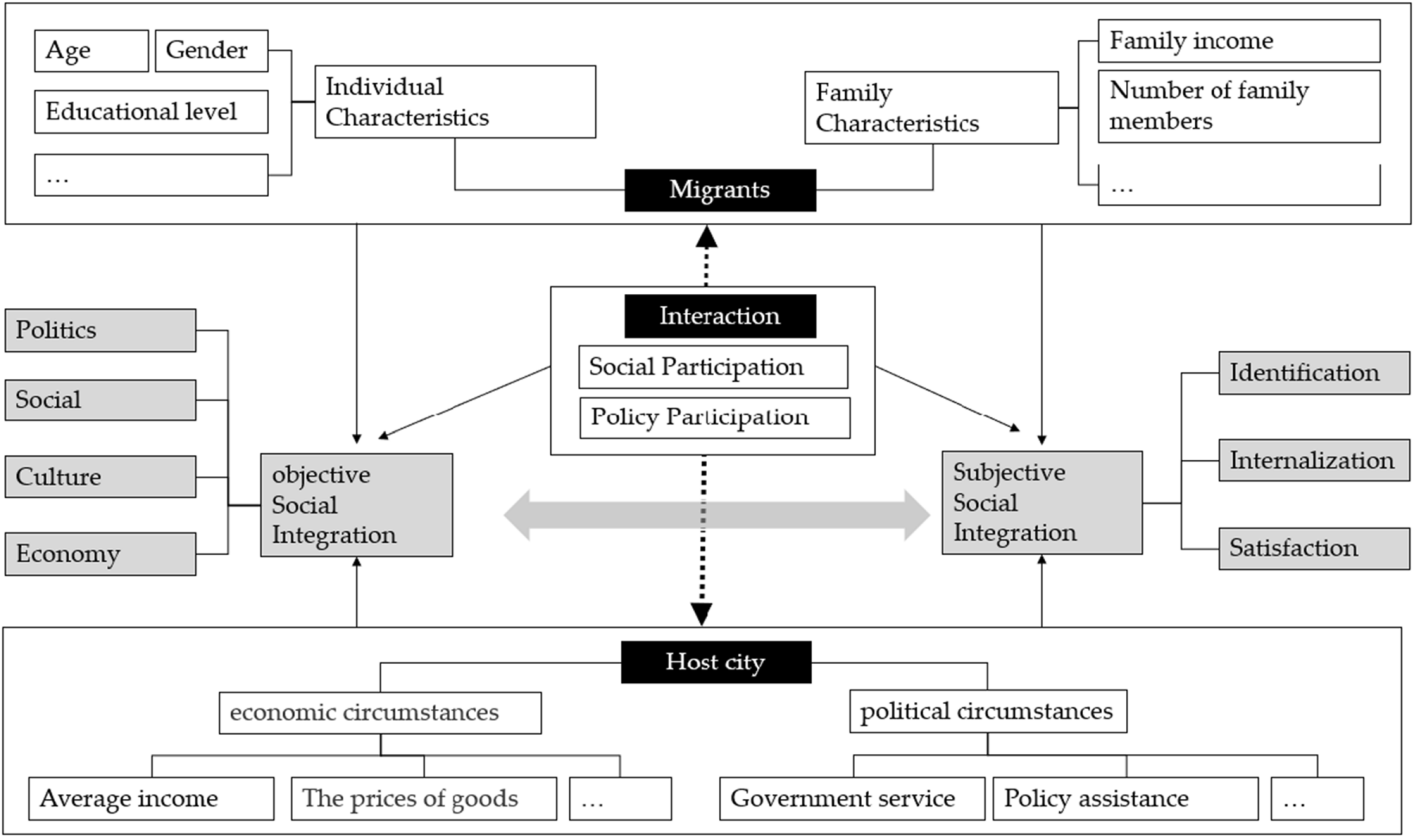
The analytical framework of social integration.

Social integration was first put forward by French sociologist Durkheim, which includes many aspects such as economy, culture, politics and psychology^8,9^. Among these, economic, political and cultural integration is objective social integration, while psychological integration is subjective social integration^10^. Subjective social integration, also known as emotional integration, belongs to the level of consciousness and is an internal social integration with hidden characteristics^11,12^. Social integration is formed by the interaction of subjective social integration and objective social integration (Fig. 1). Objective social integration evaluates the types of work, household registration and lifestyle, while subjective social integration evaluates social perception, values and identity^13,14^. Subjective social integration emphasizes the psychological integration of floating population actively integrating into the city, and individuals subjectively have the willingness to integrate, love the social environment and cultural customs of the city, and think that the good development of the city is related to themselves, so they are willing to pay attention to the inflow cities. As for the measurement of subjective social integration, the related research of migration psychology assesses the migrants' adaptation to the inflow place using the happiness and emotion (positive/negative) index^13^. Sociological research divides subjective social integration into three aspects: identification, internalization and satisfaction^15^.

At present, there are three influential schools of social integration theory: integration theory, multiculturalism theory and segmented-assimilation hypothesis^16^. Among these, the integration theory holds that migrants gradually integrate into society under the influence of urban economy and system, and integrate into the city in terms of cultural preference, identity and behavior habits^17,18^. Multiculturalism theory emphasizes individual differences, and holds that the heterogeneity of individuals and groups, along with the heterogeneity of the environment affect urban integration^19–23^. On the basis of integration theory, segmented-assimilation hypothesis explores the interactive relationship between migrants and cities^24,25^.

Among personal and family factors, age, gender, household registration, education level, marriage, family size and family income are the main factors influencing social integration^26,27^. Personal factors, such as gender, age, education and household registration, affect migrants' social role, urban cultural integration ability and labor market competitiveness, further affect migrants' cultural integration, social identity and happiness^28,29^. In addition, migrants with varying family sizes and economic incomes exhibit different lifestyles, social styles and economic pressures, so they have different integration situations^30^. The economic and political environment of the host city includes average income, commodity prices, government services, etc.^31^. A humanized policy environment can contribute to the social integration of migrants^32^. The interaction between the host city and migrants is influenced by social participation, political participation, making friends, obtaining social welfare and so on^14^. Medical insurance can improve the sense of security of migrants and reduce economic pressure. The right to participate in political activities enhances the identity of migrants during their interaction with host cities^33,34^. In the process of participating in social activities and making friends with the residents of the host city, migrants can communicate with the locals, relieve loneliness and improve happiness^35,36^.

In the existing research on the influencing factors of social integration, subjective social integration and objective social integration are mostly weighted to obtain the comprehensive social integration index. After obtaining the comprehensive social integration index, the influences of individuals, inflow places and various factors in the interaction between individuals and inflow places on social integration are calculated^9,31,37^. This ignores the difference between subjective social integration and objective social integration. According to the findings of these studies, it is difficult to distinguish the influence of various influencing factors on the subjective social integration of migrants. At present, China has experienced 40 years of urbanization, and the process of social integration of rural–urban migrants has developed from the objective level of economy, system and culture to the subjective level related to migrants' psychology. It is essential to calculate the subjective social integration index of migrants separately and analyze the influence of various influencing factors on migrants' psychology. In addition, the existing research on the influencing factors of social integration summarizes and calculates the survey data from migrants worldwide, and rarely divides migrants into regions, ignoring the spatial heterogeneity of influencing factors.

## Methodology

### Research framework

**Figure 2:**
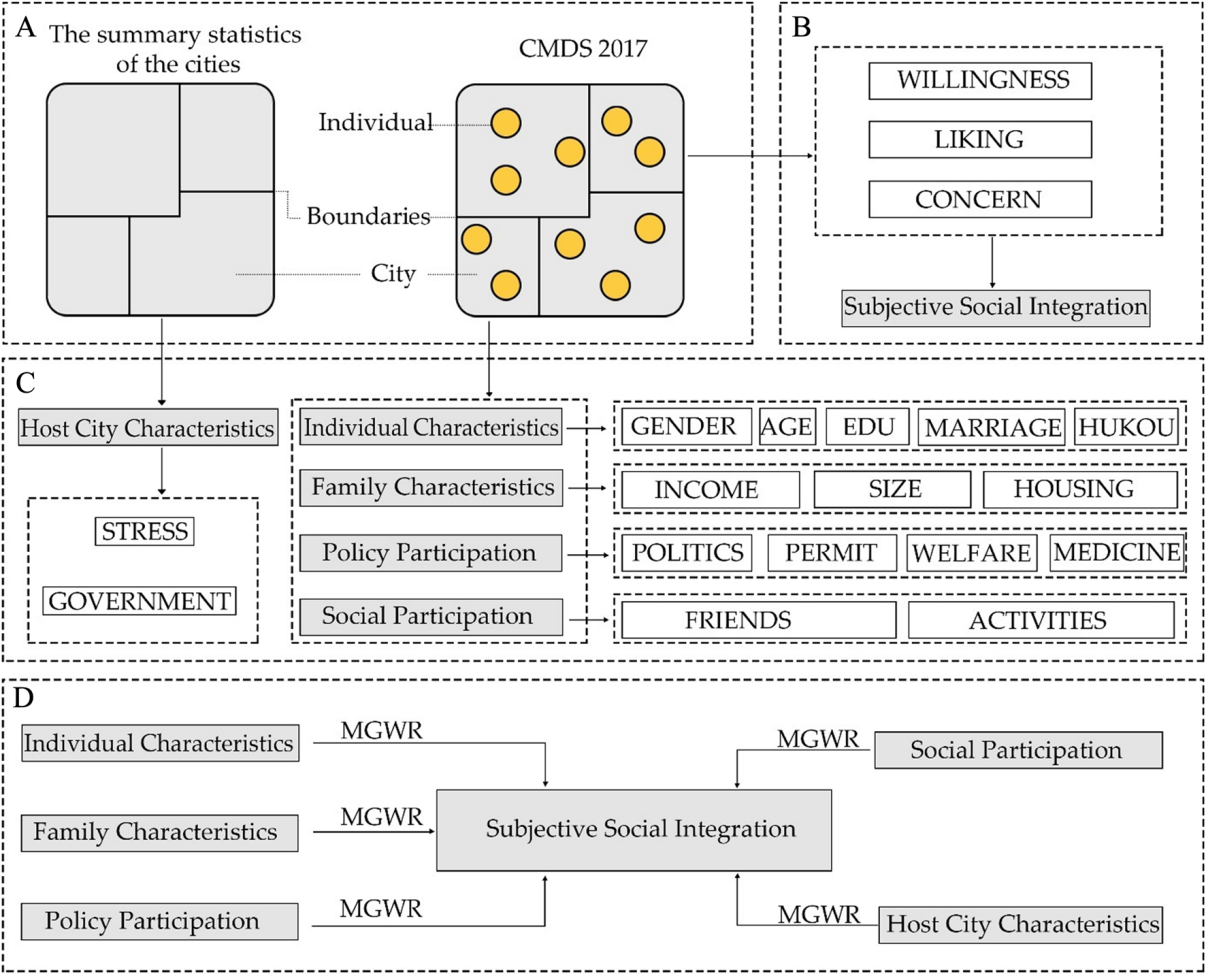
Flowchart of research: (**A**) dataset preparation, (**B**) dependent variable selection, (**C**) independent variable selection, and (**D**) model construction.

The research framework consisted of four parts, namely, dataset preparation, dependent variable selection, independent variable selection, and model construction (Fig. 2). The collected data included the summary statistics of the cities and the CMDS 2017, which provides statistics for 246 cities in China (prefecture level and above), as well as survey data for 98,045 migrants. In this case, the dependent and independent variables were extracted from the above data, and the MGWR model was built using the extracted variables.

### Dataset preparation

The CMDS 2017 and the summary statistics of the cities were collected. The summary statistics of the cities were obtained from the statistical yearbooks or statistical bulletins released by the provincial statistical departments of China, as well as government and commercial data published by the Ministry of Commerce of the People’s Republic of China, including the average sales price of commercial properties, the per capita disposable income of urban residents, and the government service ratings of cities. A total of 246 cities with complete data were retained for the study. The CMDS 2017 data came from National Health Commission of China, covering 31 provinces (autonomous regions and municipalities directly under the Central Government) and cities with relatively concentrated floating populations in the Xinjiang Production and Construction Corps. Only the surveys in 2012 and 2017 included questionnaires on social integration. Given that the data from 2012 was older, the data of CMDS 2017 was selected for analysis. The PPS method with stratification, multiple stages, and a scale ratio was adopted for sampling. Some areas inhabited by ethnic minorities and areas with incomplete statistical data coverage were removed, and a total of 98,045 migrants with complete data, including their age, household income, social security, and attitudes toward the place of entry, were retained after collation. The educational level of these 98,045 rural–urban migrants is mainly middle school, reflecting an overall low educational level, with 12.96% of the total sample of migrants with specialized education or above. The average age is about 36 years old, and 52.22% of the migrants are male. Only 17.48% of the migrants in the sample are married. The average monthly family income is about CNY 7,026. A proportion of 67.22% of the migrants have small families of 3–4 persons. While most of the migrants (65.54%) have applied for residence permits, the ownership rate of family commercial or public rental housing is low at 19.03%. Nearly half of the floating population has applied for individual social security cards, and 90.16% of the floating population has medical insurance.

### Model construction

#### Indicators description

Subjective social integration was the dependent variable in the model and was obtained by weighting three indicators—WILLINGNESS, LIKING, and CONCERN—according to the mean-squared deviation method. The degree of willingness to integrate in the host city (WILLINGNESS), the degree of liking of the host city (LIKING), and the degree of concern for the host city (CONCERN) were surveyed and scored by respondents in CMDS 2017 as an integer between 1 and 4; the higher the value, the deeper the degree. The 98,045 migrant population interviewees were categorized according to their current city of residence, and the survey data of the migrant population in each city were standardized and averaged to obtain the WILLINGNESS, LIKING, and CONCERN for each city (Table 1). Subsequently, the three indicators were then weighted by the mean-squared difference method to obtain the subjective social integration. The formula for computing the weights using the mean-squared deviation method is:

1 $${p}_{j}=\frac{1}{n}\sum_{i=1}^{n}{z}_{ij}$$

2 $${\sigma }_{j}=\sqrt{\sum_{i=1}^{n}{\left({z}_{ij}-{p}_{j}\right)}^{2}}$$

3 $${w}_{j}=\frac{{\sigma }_{j}}{\sum_{j=1}^{m}{\sigma }_{j}},$$

where *i* denotes the city, *j* denotes the indicators, *n* is the number of cities, *m* is the number of indicators, ${z}_{ij}$ is the value of indicator *j* for city *i,* ${p}_{j}$ is the mean value of indicator *j*, ${\sigma }_{j}$ is the mean-squared deviation of indicator *j*, and ${w}_{j}$ is the weight of indicator *j*.

**Table 1:** Description of subjective social integration.

Indicators	Description	Ave	Max	Min
Willingness	Average degree of integration willingness for the host city (score)	3.33	3.87	2.80
Liking	Average degree of liking for the host city (score)	3.39	3.90	2.97
Concern	Average degree of concern for the host city (score)	3.35	3.87	2.84
Subjective social integration	Mean square deviation weighted value (score)	3.36	3.83	2.91

The independent variables of the model consisted of 5 categories: individual characteristics, family characteristics, policy participation, social participation, and host city characteristics, with a total of 16 indicators (Table 2). Among these 16 indicators, except for STRESS and GOVERNMENT, which were calculated based on the summary statistics of the cities, the questions related to the other 14 indicators were all filled out by the 98,045 interviewees.

**Table 2:** Description of the indicators.

Indicators	Description	Ave	Max	Min
Individual characteristics				
Gender	The proportion of males (%)	0.52	0.74	0.35
Age	Average age (years)	37.24	48.91	28.66
Edu	Average educational level (score)	2.23	3.15	1.43
Hukou	The percentage of rural Hukou (%)	0.88	1.00	0.00
Marriage	The percentage of migrants with spouse (%)	0.84	1.00	0.45
Family characteristics				
Income	Average household income (yuan)	6281.63	10,940.63	1756.67
Size	Average number of family members	3.28	4.29	2.29
Housing	The percentage of household stable housing (%)	0.25	1.00	0.00
Policy participation				
Politics	Number of times participate in political activities a year	5.79	9.41	5.06
Permit	The percentage of migrants with residence permit (%)	0.52	0.98	0.00
Welfare	The percentage of migrants with social security card (%)	0.45	0.94	0.00
Medicine	The percentage of migrants with medical insurance card (%)	0.90	1.00	0.45
Social participation				
Friends	The percentage of migrants with friends in host city (%)	0.48	1.00	0.08
Activities	Number of times participate in social activities a year	0.67	3.03	0.00
Host city characteristics				
Stress	Average house selling price (yuan/m^2^)/ per capita disposable income (yuan/year)	0.19	0.91	0.08
Government	Political and business environment (score)	19.65	100.00	1.11

Individual characteristics included the proportion of male migrants in a city (GENDER), average age (AGE), average educational level (EDU), the percentage of migrants’ rural hukou (HUKOU), and the percentage of migrants with a spouse (MARRIAGE). The educational level of migrants was categorized as "Primary Education ", "Junior Secondary Education ", "Senior Secondary Education ", or "Higher Education ", assigned as "1", "2", "3", or "4 ", respectively. Respondents were categorized based on their current city of residence in order to calculate the average educational level of migrants in each city. Family characteristics included average household income in the city (IN-COME), average number of migrant family members (SIZE), and proportion of the floating population whose families own stable housing (HOUSING). Policy participation included the percentage of migrants with a residence permit (PERMIT), the percentage of migrants with a social security card (WELFARE), and the percentage of migrants with a medical insurance card (MEDICINE). These three indicators evaluate the migrants’ access to Policy Participation according to four aspects: political activities, residence permit, social security, and medical security. Social participation included the percentage of migrants with friends in the host city (FRIENDS), participation in social activities (ACTIVITIES), and political participation (POLITICS). Participation in social activities is measured by the average number of times migrants participate in social activities in the city where they live within one year. POLITICS is also measured by the average number of times migrants participate in political activities in the city where they live within one year. Host City Characteristics include the stress of buying a house (STRESS) and government services (GOVERNMENT). Among these, GOVERNMENT is a comprehensive index, which evaluates the tax, business environment, government integrity and government transparency of the host city. The higher the value, the healthier the political and business environment of the city. Due to the considerable differences in the economic conditions of cities, the unit price of commercial housing in cities cannot reflect the pressure on migrants to buy houses. Therefore, the ratio of the unit price of commercial housing to the annual income is used to measure the housing pressure; the higher the value, the greater the pressure.

#### Global model

The ordinary least squares (OLS) model is widely used to assess the relationship between multiple independent variables and a dependent variable, with its effect on the dependent variable judged by the value of the coefficient corresponding to the inde-pendent variable^38^. In this study, we used OLS to construct a global model and calculated the influence of the independent variables on the subjective social integration at the global level. The formula is as follows.

4 $${y}_{i}={\beta }_{0}+{\sum }_{k=1}^{k=n}{\beta }_{j}{x}_{ij}+{\varepsilon }_{i} ,$$

where *i* denotes the city, ${x}_{ij}$ denotes the value of indicator j for city *i*, ${y}_{i}$ is the subjective social integration index of city *i*, ${\varepsilon }_{i}$ is the residual term, ${\beta }_{j}$ is the regression coefficient of indicator *j*, and n is the number of indicators included in the model.

#### Multiscale geographically weighted model

Compared to global regression models, geographically weighted regression (GWR) models take spatial pattern elements into account and are therefore widely used to test for spatial heterogeneity effects, typically a providing higher model fit and a lower residual spatial autocorrelation^39^. MGWR is an extension of the GWR framework that allows each relationship in the model to vary at a unique spatial scale. MGWR is much less restrictive in its assumptions than GWR, as the relationships between responses and covariates are allowed to vary locally, regionally, or not at all^40^. MGWR can calculate regression coefficients for each study unit and select the appropriate bandwidth to calculate regression coefficients depending on the scale of action of the indicators. The multiscale inference methods used by the model obtained reliable local parameter estimates that allowed for quantitative and local analysis of the spatial heterogeneity of the forces affecting the subjective social integration in each city, and the scale of influence of each type of factor on the subjective social integration was calculated^41,42^. The model was constructed as follows.

5 $${y}_{i}={\sum }_{j=1}^{k}{\beta }_{bwj}{({u}_{i},{v}_{i})x}_{ij}+{\varepsilon }_{i},$$

where *i* is the city, *j* is the indicator, ${x}_{ij}$ is the value taken for indicator *j* of city *i*, $bwj$ denotes the bandwidth used for the regression coefficient of indicator *j*, ${\beta }_{bwj}$ is the regression coefficient of indicator *j*, and $({u}_{i},{v}_{i})$ represents the spatial location of city *i*.

## Results

### Spatial distribution of the subjective social integration

**Figure 3:**
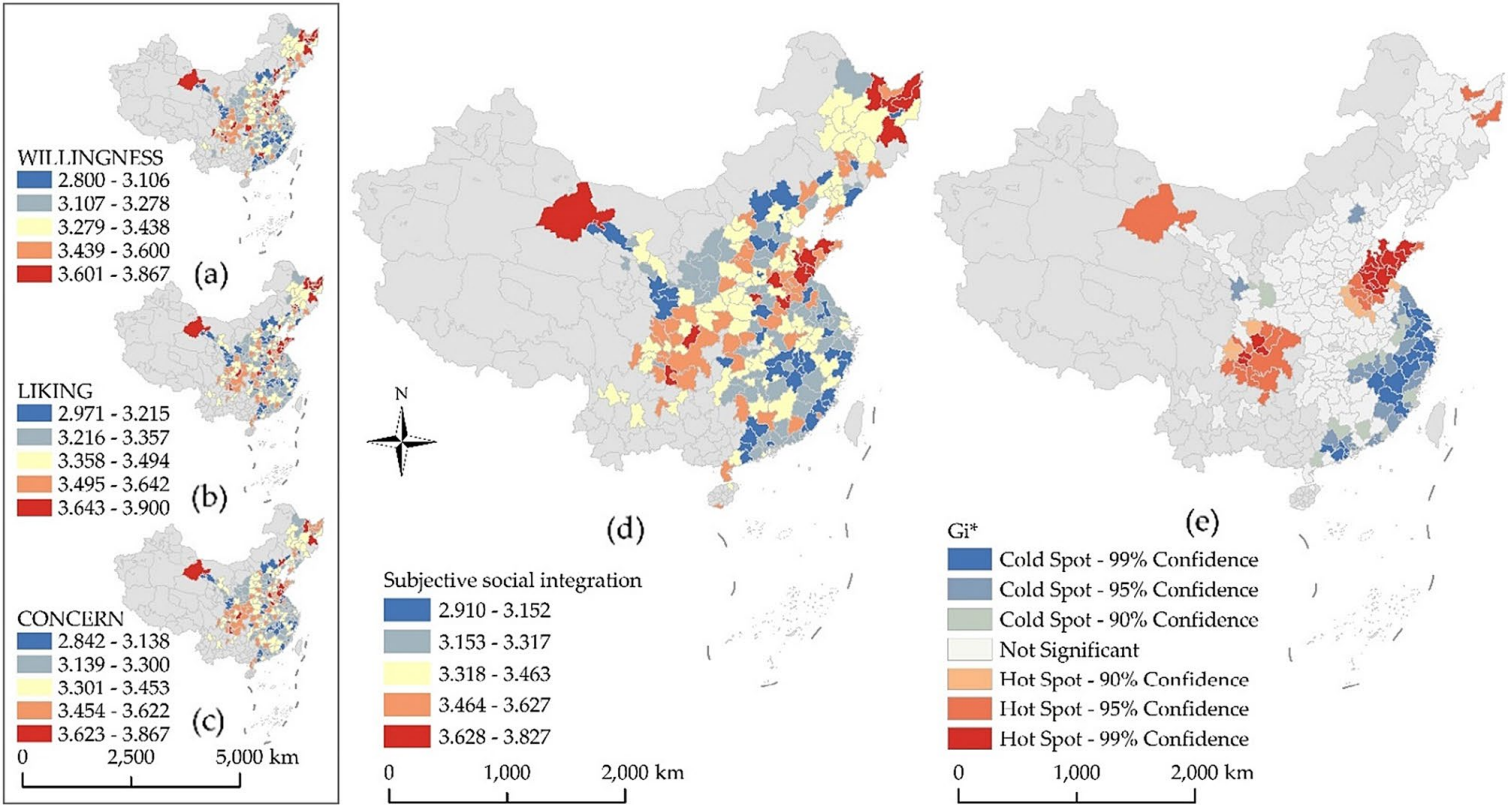
Spatial distribution of WILLINGNESS (**a**), LIKING (**b**), and CONCERN (**c**). Spatial distribution (**d**) and Getis-Ord Gi* (**e**) of the subjective social integration index.

To visually present the spatial characteristics of the subjective social integration of the floating population in China, 246 cities were divided into five grades using the natural breakpoint method. Figure 3a–c shows the three subitems that constitute the subjective social integration index, assessing the migrants’ willingness to integrate into society, their love for the inflow cities, and their concern for the inflow cities. The values range between 1 and 4, and the larger the value, the deeper the measure. The three dimensions of subjective social integration are compared in Table 2 and Fig. 3a–c. The integration level of each dimension has similar spatial distribution characteristics, with dimension scores, in order from high to low, being LIKING > CONCERN > WILLINGNESS, which shows that the development of subjective social integration of migrants is hierarchical. They like the inflow city first, then care about the inflow city, and finally have a willingness to integrate.

The subjective social integration index, obtained using the mean-squared deviation method, ranges from 1 to 4. The larger the value, the higher the degree of subjective social integration of urban migrants. To visually present the spatial characteristics of the subjective social integration of Chinese migrants, similarly, 246 cities were divided into five grades using the natural breakpoint method, as shown in Fig. 3d. From a regional perspective, there are considerable differences in the level of subjective social integration of migrants in cities. The level of social integration in southeast coastal cities is generally low, whereas the level of subjective social integration of the Shandong Peninsula urban agglomeration is high.

**Figure 4:**
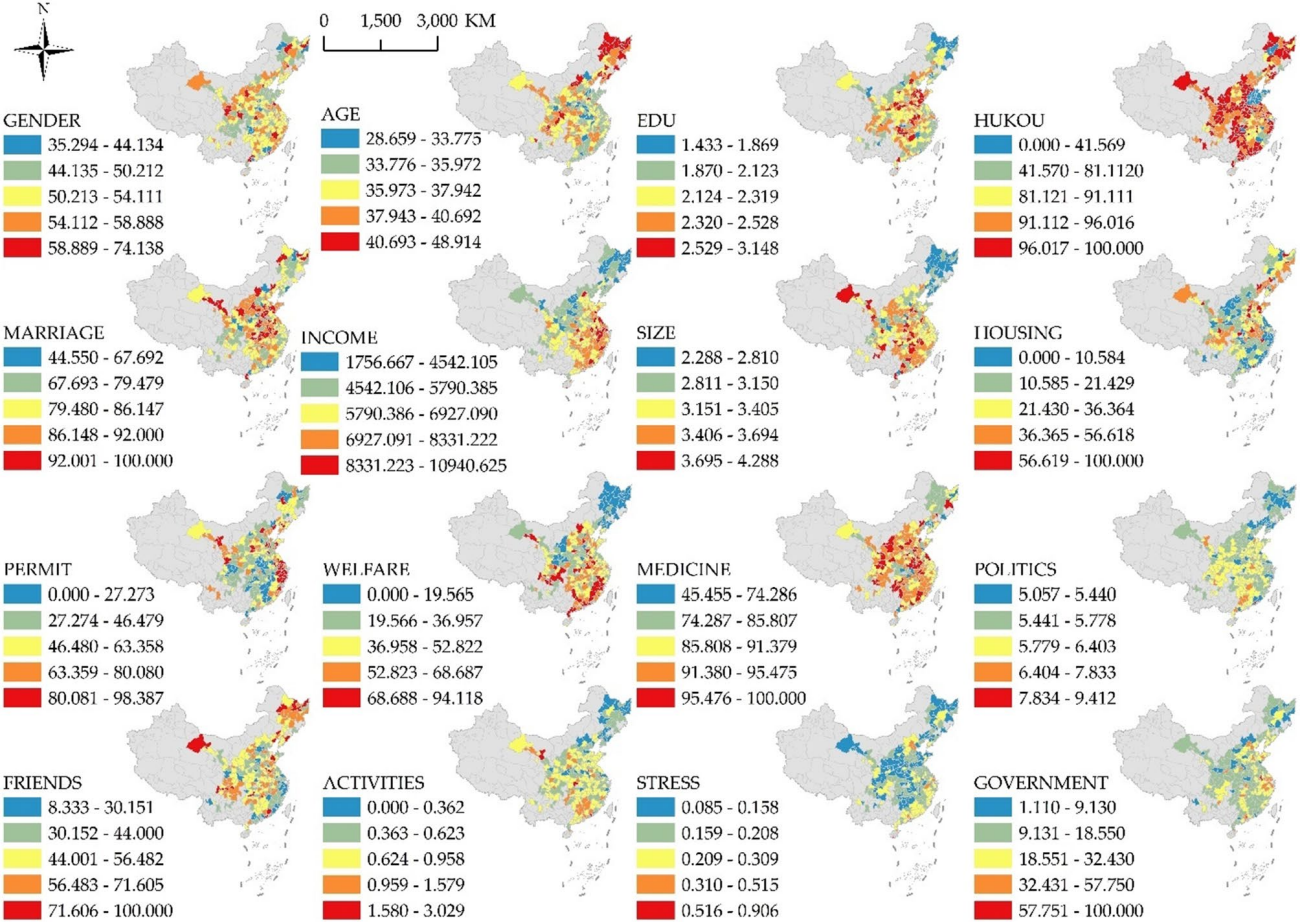
Spatial distribution of the indicators.

To analyze the spatial pattern of the subjective social integration index more intuitively, spatial autocorrelation analysis was used to calculate the dependent variable. Global spatial autocorrelation was first applied to test for a spatial autocorrelation; then hot and cold spots were calculated via local spatial autocorrelation (Getis-Ord Gi*). The global Moran I index was 0.24, with a z-score of 7.97 (exceeding the critical value of 2.56) and a p-value of less than 0.01, indicating that the subjective social integration index of the urban migrant population was spatially correlated^43^. Further local spatial autocorrelation (Getis-Ord Gi*) was conducted to identify high- and low-value agglomeration areas (Fig. 3e). Two high-value agglomerations were identified, namely Chengdu–Chongqing urban agglomeration and its surrounding cities, and east–central Shandong and its surrounding cities, whereas the low-value agglomerations were concentrated mainly along the southeastern coast. As shown in Fig. 4, for Shandong Peninsula urban agglomeration, the proportion of female migrants in each city is high (above 50%), the marriage rate of migrants is high (above 80%), and the rural household registration is low (below 40%), which may be the reasons for the high-value agglomeration of subjective social integration in this area. The southeastern coastal area of China, exhibiting low cluster values in the subjective social integration index, is the earliest developed area in China after the Reform and Opening-up and is the region with the highest rural-to-urban migrant population concentration in China. For the southeast coastal areas, the proportion of men is relatively high (generally higher than 50%), and the proportion of migrants who socialize with local people is low (below 45%), which may be the reason for the low-value agglomeration of subjective social integration in this area.

### Model comparisons

In this study, we first ran an OLS model that provides global benchmark test results and preliminarily verified an obvious causal correlation between the five types of influencing factors above and the subjective social integration index. Then, the spatial heterogeneity of influencing factors was explored at the local scale using the GWR model. However, the GWR model cannot identify scale differences of factors. To reveal the spatial non-stationarity between different factors and dependent variables, the MGWR model is constructed, which allows each variable to have different bandwidths. According to the fitting effect of the three models (Table 3), the adjusted R2 of the MGWR model is 0.581, and the AICc value is lower than that of the other two models. From the perspective of bandwidth difference, the bandwidth of all independent variables in the GWR model is 214, whereas the bandwidth of variables in MGWR model vary, indicating that the relationship between their respective variables and dependent variables is spatially heterogeneous.

**Table 3:** Model comparisons.

	Model 1: OLS		Model 2: GWR			Model 3: MGWR	
	β(SE)		$\overline{\upbeta }$($\overline{{\text{SE}} }$)	Bandwidths		$\overline{\upbeta }$($\overline{{\text{SE}} }$)	Bandwidths
Gender	− 0.141** (0.058)		− 0.143* (0.068)	214		− 0.153** (0.056)	241
Age	0.076 (0.083)		0.042 (0.098)	214		0.021 (0.087)	236
Edu	0.112 (0.080)		0.110 (0.101)	214		0.012 (0.179)	46
Hukou	− 0.156*** (0.059)		− 0.121* (0.066)	214		− 0.106* (0.063)	235
Marriage	− 0.094 (0.069)		− 0.151 (0.084)	214		− 0.149 (0.093)	145
Income	− 0.245*** (0.074)		− 0.228*** (0.085)	214		− 0.119 (0.079)	245
Size	− 0.016(0.073)		− 0.119 (0.094)	214		− 0.107 (0.077)	245
Housing	0.167** (0.065)		0.179* (0.082)	214		0.170 (0.99)	131
Politics	0.233*** (0.079)		0.286** (0.104)	214		0.313** (0.102)	173
Permit	0.094 (0.064)		0.132 (0.079)	214		0.147 (0.075)	205
Welfare	− 0.050 (0.064)		− 0.027 (0.078)	214		0.025 (0.071)	223
Medicine	0.020 (0.058)		0.005 (0.073)	214		0.007 (0.057)	245
Friends	0.223*** (0.069)		0.150 (0.085)	214		0.127 (0.124)	83
Activities	0.029(0.080)		0.084 (0.098)	214		0.093 (0.076)	245
Stress	0.072 (0.070)		0.051 (0.102)	214		0.059 (0.072)	245
Government	0.014 (0.074)		− 0.052 (0.093)	214		− 0.030 (0.071)	245
Intercept	− 0.000(0.052)		− 0.006(0.069)	214		− 0.016 (0.126)	72
*R*^2^	0.366		0.506			0.619	
Adj. *R*^2^	0.322		0.417			0.514	
AICc	624.981		615.717			598.862	
Log-likelihood	− 292.984		− 262.378			− 230.463	

*Significant at the 0.1 level.

**Significant at the 0.05 level.

***Significant at the 0.01 level.

According to the MGWR model, out of the 16 indicators that affect subjective social integration, 6 are global indicators, namely: INCOME, SIZE, MEDICINE, ACTIVI-TIES, STRESS, and GOVERNMENT, and are suitable for measuring influence across the whole country. The remaining ten indicators are suitable for analysis on a local scale. Among these, EDU and FRIENDS have bandwidths of 46 and 72, respectively, as well as strong spatial heterogeneity, and their influence on subjective social integration changes considerably with regional variations. HOUSING, MARRIAGE, and POLITICS belong to the middle scale, with bandwidths of 131, 145, and 173, respectively. The bandwidth values of the other indicators are all greater than 200; these indicators are spatially heterogeneous but weak, and their influence changes little with regions variations. What needs to be further pointed out is INCOME, which has a significant negative effect in OLS model. However, in the MGWR model, while it also exhibits a negative effect, it is not significant. This is because MGWR model considers geographical differences, so some local indicators that are not significant in OLS model decrease in some cities, which improves the average *P* value of global variables.

### The global benchmark results

The OLS results of six global indicators (Table 3) indicate that an increase in family income significantly reduces the subjective social integration of migrants. The average family income of migrants in a city reflects the economic development level of the city, and subjective social integration reflects the migrants’ willingness to identify, contact, and integrate into the city. Cities with a high level of economic development, such as Beijing, Shanghai, and Guangzhou, have a fast pace of life, high consumption levels, and difficulty in obtaining an urban hukou. Therefore, migrants have low urban identity, weak contact with cities, and low willingness to integrate, which, in turn, affect subjective social integration.

### Mechanism for spatial heterogeneity

**Figure 5:**
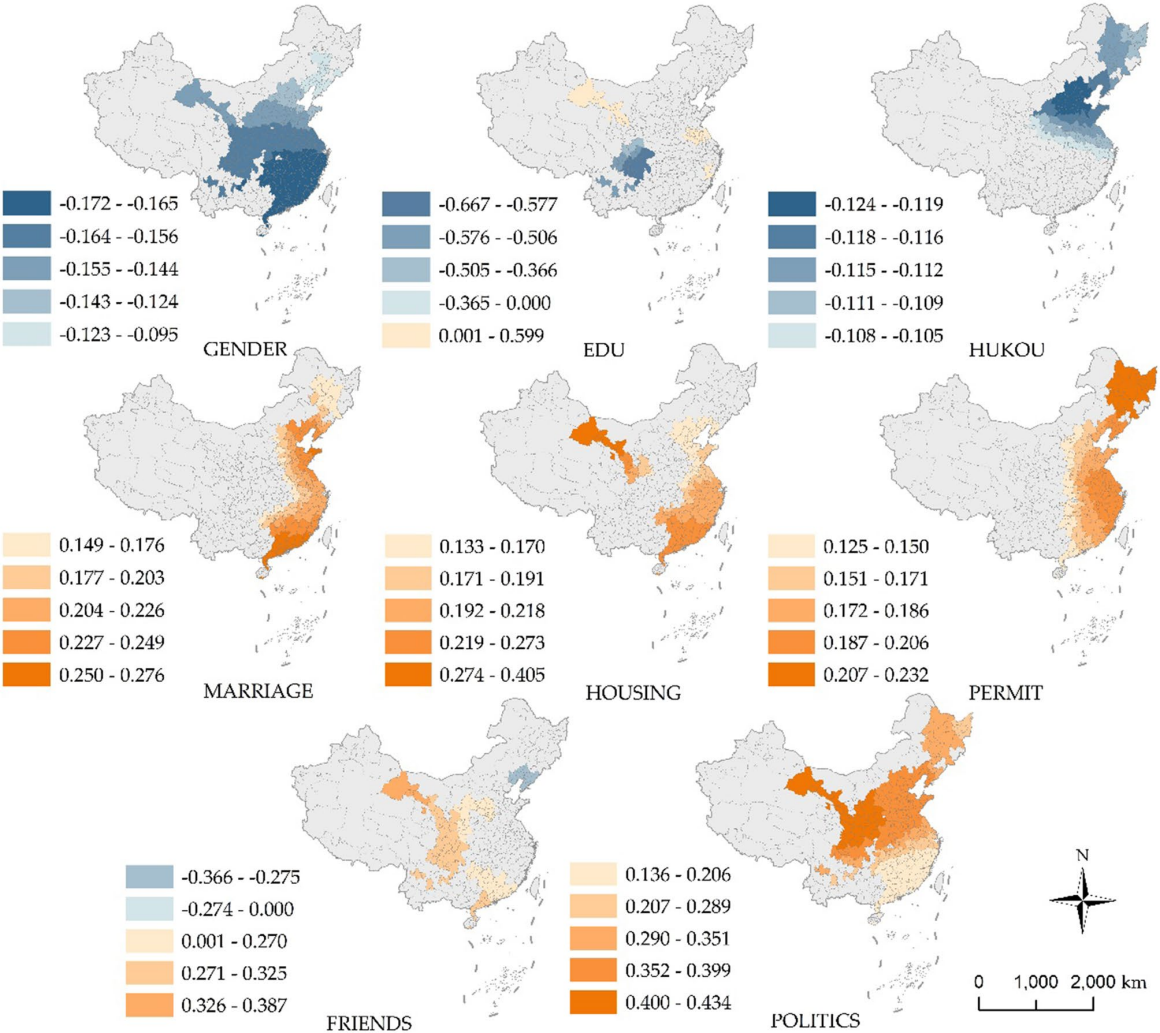
Spatially divergent patterns of subjective social integration impact indicators.

The MGWR model calculates the coefficient differentiation of the same variable in different cities through local regression. In this study, only the variables satisfying a significance of p < 0.1 were selected. According to the classification of natural breakpoints, cities were divided into five categories, reflecting the spatial differences in the degree of influence of non-stationary variables in each space, as shown in Fig. 5.

Among the individual characteristics, GENDER, EDU, HUKOU, and MARRIAGE have a significant influence on subjective social integration and exhibit spatial heterogeneity. The impact of GENDER on subjective social integration is negative, with the regression coefficient showing a south–north differentiation pattern. According to the statistics, in 73% of cities, the proportion of the male floating population is higher than that of the female floating population. Therefore, in cities with a higher proportion of male floating population, the gender imbalance of the floating population is more serious. The imbalance between men and women makes it more difficult for migrants to choose a spouse, and the increase in the single rate reduces migrants’ sense of belonging to the city, thereby reducing their subjective social integration. The influence of EDU on subjective social integration exhibits a strong spatial heterogeneity, and the educational level in Sichuan and Chongqing has a significant negative effect on subjective social integration, whereas the opposite is true for central and northern Gansu. The influence of HUKOU on subjective social integration has a negative effect, with the Beijing–Tianjin–Hebei urban agglomeration as the core and decreasing towards the outer circle. The smaller the proportion of rural hukou migrants in the inflow cities, the less the proportion of migrants who have obtained an urban hukou. In China, migrants without an urban hukou are restricted in many aspects, such as the children’s education, social welfare, medical security, and participation in political activities, so the degree of subjective social integration of rural hukou migrants is generally low. In the Beijing–Tianjin–Hebei urban agglomeration, especially in Beijing, the political center of China, rural migrants experience difficulties in obtaining an urban hukou. Whether they can settle down has a greater influence on the subjective social integration of migrants than in other regions. MARRIAGE has a positive effect on subjective social integration, and the coefficient shows a coastal–inland differentiation pattern. The increase in the proportion of married migrants in the inflow cities can promote the subjective social integration of these cities. The subjective social integration of coastal cities is more influenced by MARRIAGE than inland areas, especially the Pearl River Delta urban agglomeration.

Among the indicators related to family characteristics, HOUSING has a positive effect on subjective social integration, and the regression coefficient shows a different pattern from southwest to northeast. The greater the proportion of migrants with stable housing (self-purchased houses or public rental houses) in the inflow cities, the higher the degree of subjective social integration of the cities, with decreasing influence from southwest to northeast. On one hand, migrants have stable housing in the inflow city, on the other hand, they increase their sense of belonging to the city and strengthen their ties with the city, thereby enhancing the degree of subjective urban integration.

From the perspective of policy participation, PERMIT has a positive effect on subjective social integration, and the regression coefficient shows an east–west differentiation pattern. The increase in the proportion of the floating population who have applied for a residence permit is conducive to the improvement of subjective social integration. Because migrants who apply for residence permits can more conveniently apply for settlement, motor vehicle licenses, and government housing subsidies, these conveniences can enhance the happiness of migrants and thereby enhance subjective social integration.

Among the indicators related to Social Participation, FRIENDS and POLITICS have a positive effect on subjective social integration. Among these, POLITICS has a considerable influence, and the regression coefficient shows a pattern of north–south differentiation, whereas FRIENDS has little influence and exhibits a high level of spatial heterogeneity. The average number of times migrants participate in political activities reflects the tolerance of migrants in the cities where they flow, and participation in political activities signifies the acceptance of migrants. The force of this influence increases from southeast to northwest.

## Discussion and conclusions

### Discussion

In this article, we analyzed the influence of individual characteristics, family characteristics, policy participation, social participation, and other factors on the subjective social integration of migrants. The biggest difference from previous studies is that our MGWR study effectively reveals the spatial heterogeneity of the influencing factors. This work has tangible policy guidance value and can support the promotion of migrant integration into cities and urbanization, as the results illustrate.

We begin with a series of recommendations for Northern China, given the prominent influence of certain factors. Northern China should accelerate the reform of the household registration system to increase the willingness of the floating population to settle in cities. Nowadays, all but a very few mega-cities should completely liberalize settlement restrictions. Finally, local governments should appropriately ease the voter eligibility restrictions and promote the participation of the floating population in urban management and decision-making, which is conducive to enhancing their subjective social integration. Due to cultural differences between the north and the south of China, the north is more influenced by the official-oriented ideology and has higher requirements for political participation.

For Southern China, housing has become a more important and urgent issue. The most important thing is to solve the housing shortage, improve the supply of public rental housing in cities, and pay attention to the negative impact of high housing prices in big cities on subjective social integration. A significant portion of the migrant population needs to purchase a home to obtain stable residency in the city. However, high housing prices in cities increase the cost of home ownership for the migrant population and reduce their willingness to settle down and start a family, resulting in the migrant population not developing a strong sense of belonging to such a city. Secondly, Southern China should further build policies for gender-friendly cities for migrants. The overall migration of families promotes the degree of subjective social integration, and therefore it is necessary to provide corresponding family assistance policies so that each individual can live a better life. This also requires the provision of corresponding, adequate, and caring jobs for females. Of course, parts of the South also have to address issues related to marriage and friend-making. The abundance of enterprises in the South (as well as in Eastern China) creates a closed "factory society" that limits people's socialization needs and the possibility of marriage, which requires more effective policies in terms of land-use layout and communication. For example, adding services and recreational facilities to an industrial area, or developing cross-business exchange activities could improve the situation.

Certainly, it is not the case that there are no crucial concerns elsewhere. We also find that Northeast China is most sensitive to permits, and Northwest China is similarly affected by housing and labor skills. We would also like to emphasize that some factors, such as income, welfare, and healthcare, are indiscriminately sweeping across most of China. These are unavoidable influences. In addition, some drawbacks are that we did not investigate several mediating factors that do not directly affect subjective social integration. Therefore, in future research, we will attempt to introduce a structural equation model (SEM) to analyze the interaction between indicators in order to deeply analyze the mechanism of subjective social integration.

### Conclusions

In this study, based on the data of CMDS 2017, the researcher used MGWR model to analyze the influencing factors of subjective social integration and the spatial heterogeneity of these factors. The subjective social integration of migrants is obtained by weighted calculation of WILLINGNESS, LIKING and CONCERN. This article constructs an analytical model for influencing factors from three aspects: migrants, host cities and the interaction between migrants and host cities, and divides them into five types of influencing factors and 16 indicators.

This study differs from previous studies on social integration as it focuses on the psychological level of migrants and studies subjective social integration. The research innovatively introduces MGWR model to examine the influencing factors of subjective social integration, categorizing them into 6 global indicators and 10 local indicators. This approach solves the problem that the traditional OLS model cannot simulate the spatial differences of influencing factors. The main contribution of the study is to clarify the dominant factors in different regions through the spatial heterogeneity analysis of influencing factors, so as to put forward differentiated suggestions for the adjustment of immigration policy.

From the spatial pattern of subjective social integration, there is spatial autocorrelation in the subjective social integration of rural–urban migrants. The low-level integration area is mainly located in the southeast coastal areas, while the high-level integration area is mainly found in the Shandong Peninsula urban agglomeration and Chengdu-Chongqing urban agglomeration. The development of migrants' subjective social integration is hierarchical. Migrants initially exhibit emotional preference for the inflow cities, then pay attention to the development of the inflow cities, and finally are willing to integrate into the inflow cities.

Among the influencing factors, gender, household registration, participation in political activities, housing and making friends have a significant impact on the subjective social integration of migrants. As for the spatial heterogeneity of influencing factors, the influence of household registration and participation in political activities in northern China is higher than that in southern China. The northern region should accelerate the reform of household registration system and ease the restrictions on voter qualifications. Housing and marriage have a greater impact on southern China. For the southern region, especially the Pearl River Delta urban agglomeration and the eastern coastal areas, accelerating the improvement of gender-friendly policies, reducing the pressure on migrants to buy houses, improving the supply of urban public housing, promoting the social interaction of migrants and increasing the marriage rate of migrants can more effectively enhance the subjective social integration of migrants (Supplemenrtary Information).

### Supplementary Information

Supplementary Information.

## Data Availability

Publicly available datasets were analyzed in this study. CMDS 2017 can be found here: [https://chinaldrk.org.cn/wjw/#/home]. The datasets used and/or analyzed during the current study are available from the corresponding author on reasonable request.
