# Supplementary material for: Subjective social integration and its spatially varying determinants of rural-to-urban migrants among Chinese cities
Source: Sci Rep. 2024 Mar 6;14:5540. doi: 10.1038/s41598-024-55129-y (PMC10918073; doi:10.1038/s41598-024-55129-y)
Supplement: Supplementary file 1 — Supplementary Information. [file 41598_2024_55129_MOESM1_ESM.pdf]

ID	x_coordne	y_coordne	Subjective social integration	GENDER	AGE	SKILL	HUKOU	MARRIAGE	INCOME	SIZE	HOUSING	PERMIT	WELFARE	MEDICINE	FRIENDS	ACTIVITIES	POLITICS	STRESS	GOVERNMENT
225	121.9552	42.27926	2.910249	0.526316	37.57895	2.157895	1	0.842105	4542.105	3.315789	0.526316	0.736842	0.263158	0.894737	0.736842	0.526316	5.157895	0.14428	17.03
155	116.7855	23.78555	2.957603	0.555556	35.05556	1.833333	0.972222	0.861111	8652.778	3.555556	0	0.277778	0.222222	0.944444	0.083333	0	5.083333	0.258647	1.11
1	130.9359	45.87901	2.991579	0.514286	41.57143	1.714286	1	0.885714	4014.286	2.742857	0.857143	0.142857	0.057143	0.628571	0.457143	0.342857	5.6	0.16661	15.45
17	111.7937	22.81603	2.994488	0.6	36.62857	2.028571	0.971429	0.914286	5891.886	3.628571	0.057143	0.542857	0.571429	0.971429	0.142857	0.657143	6.057143	0.208266	2.18
90	99.8227	38.95579	3.009852	0.461538	38.88462	2.076923	0.923077	0.961538	5403.846	3.230769	0.230769	0.769231	0.807692	0.769231	0.5	1.230769	5.615385	0.147154	16.42
56	105.738	34.64723	3.01155	0.611111	34.36111	2.166667	0.916667	0.805556	5947.222	3.083333	0.25	0.611111	0.305556	0.888889	0.416667	0.277778	5.333333	0.241063	1.67
105	117.547	41.34721	3.015608	0.587302	33.40476	2.357143	0.952381	0.634921	5438.095	3.34127	0.126984	0.31746	0.492063	0.904762	0.507937	0.18254	5.301587	0.216441	6.9
37	121.1372	28.75727	3.025122	0.555766	35.69943	1.924386	0.964083	0.746692	6924.384	3.268431	0.011342	0.858223	0.340265	0.913043	0.175803	0.345936	5.383743	0.176529	24.45
221	119.4528	32.01572	3.029849	0.492537	36.41791	2.119403	0.970149	0.761194	6132.836	3.089552	0.014925	0.522388	0.358209	0.910448	0.268657	0.970149	5.343284	0.187987	30.63
100	116.4341	27.51015	3.030888	0.464286	38.39286	1.767857	0.910714	0.910714	7555.893	3.464286	0	0.178571	0.232143	0.892857	0.196429	0.410714	5.732143	0.17493	12.66
144	120.4635	27.89587	3.034162	0.55662	36.28049	1.91115	0.978223	0.845819	7544.497	3.536585	0.010453	0.961672	0.422474	0.892857	0.258711	0.689895	5.5	0.237227	23.21
241	117.1074	28.22464	3.04752	0.548077	37.625	2	1	0.913462	6768.269	3.644231	0.192308	0.25	0.423077	0.990385	0.432692	0.75	5.865385	0.149137	21.19
226	111.7746	22.02783	3.048308	0.428571	34.53571	2.142857	0.964286	0.821429	6042.857	3.714286	0.214286	0.107143	0.821429	0.892857	0.428571	0.678571	5.142857	0.198925	14.31
214	119.9522	29.11711	3.05826	0.530777	34.49647	2.059536	0.9556	0.722503	7686.408	3.18668	0.010091	0.887992	0.479314	0.889001	0.246216	0.432896	5.43996	0.222257	36.06
175	112.2053	23.53771	3.06384	0.514563	37.24272	1.961165	0.990291	0.864078	7598.058	3.728155	0.126214	0.563107	0.543689	0.912621	0.174757	0.699029	6.815534	0.216225	13.48
74	118.5202	33.78426	3.072917	0.5625	37.5	2.28125	0.9375	0.84375	10940.63	3.75	0.21875	0.53125	0.34375	0.875	0.65625	0.65625	5.84375	0.184777	18.04
181	118.9001	25.44362	3.07634	0.504545	35.72727	1.904545	0.809091	0.813636	7812.727	3.5	0	0.727273	0.527273	0.927273	0.231818	0.818182	5.509091	0.250913	16.3
87	116.6283	39.25938	3.087096	0.602201	37.41509	2.056604	0.970126	0.746855	6517.296	2.834906	0.073899	0.427673	0.183962	0.944969	0.246855	0.397799	5.371069	0.324171	20.05
111	117.27	29.30728	3.088023	0.519685	37.70866	2.031496	0.984252	0.905512	7477.945	3.889764	0.204724	0.330709	0.440945	0.952756	0.409449	0.566929	5.606299	0.142141	16.32
70	114.9726	28.30615	3.095206	0.560345	37.16379	2.353448	0.887931	0.913793	6434.483	3.732759	0.155172	0.37069	0.508621	0.913793	0.681034	0.663793	5.862069	0.15932	16.6
189	115.8226	37.7625	3.096567	0.494624	39.44086	2.172043	0.978495	0.548387	5966.667	2.55914	0.139785	0.139785	0.860215	0.978495	0.376344	0.419355	5.451613	0.183203	14.54
5	117.4722	28.77295	3.096619	0.504545	34.38182	2.263636	0.863636	0.836364	7794.318	3.709091	0.181818	0.218182	0.313636	0.95	0.677273	0.654545	5.704545	0.167928	19.42
152	117.4539	24.33173	3.101176	0.547872	35.60106	1.968085	0.984043	0.765957	6090.957	3.473404	0.031915	0.569149	0.664894	0.904255	0.287234	0.414894	5.388298	0.217393	15.99
89	110.5266	29.39246	3.109825	0.466667	39.66667	2.366667	0.966667	0.733333	6060	3.9	0.366667	0.3	0.6	0.9	0.266667	0.6	5.2	0.192555	1.61
11	124.3974	40.54001	3.110446	0.538462	42.8	1.907692	1	0.676923	4690.769	2.569231	0.430769	0.523077	0.046154	0.876923	0.738462	0.292308	5.292308	0.170877	19.42
239	114.3092	35.72324	3.110615	0.615385	38.17949	2.051282	1	1	5435.897	3.589744	0.076923	0.717949	0.487179	1	0.179487	0.153846	5.307692	0.150387	19.43
6	113.875	22.93453	3.113302	0.513202	28.65945	2.436019	0.974949	0.445498	6039.477	2.288422	0.019634	0.576845	0.7109	0.821259	0.352742	0.987136	5.856466	0.36109	100
85	115.0318	40.86491	3.114536	0.468571	41.12571	2.017143	0.965714	0.942857	4731.429	3.234286	0.171429	0.348571	0.777143	0.942857	0.537143	0.217143	5.457143	0.2252	4.9
229	105.2219	33.59691	3.114804	0.606742	37.65169	2.022472	0.932584	0.898876	7737.978	3.595506	0.05618	0.94382	0.438202	0.921348	0.258427	0.505618	5.516854	0.212124	11.15
68	104.4532	35.11962	3.115995	0.582524	37.94175	2.029126	0.951456	0.893204	6603.883	3.970874	0.174757	0.68932	0.407767	0.902913	0.495146	0.796117	6.097087	0.179035	12.39
133	120.0563	32.571	3.127486	0.588889	35.02222	2.238889	0.95	0.783333	6997.222	2.961111	0.05	0.833333	0.516667	0.866667	0.494444	0.327778	5.777778	0.177963	17.36
169	114.4395	38.13053	3.131412	0.565668	35.48042	2.454493	0.973502	0.756912	5554.671	2.91129	0.164171	0.641129	0.537442	0.940668	0.385945	0.645161	5.698733	0.308846	28.12
143	112.875	24.31431	3.133537	0.568421	33.97895	2.494737	0.957895	0.778947	7662.105	3.021053	0.052632	0.431579	0.736842	0.926316	0.442105	1.010526	6.757895	0.235313	14.68
16	115.4517	29.32197	3.134162	0.570093	36.51869	2.350467	0.943925	0.836449	6255.14	3.271028	0.158879	0.434579	0.565421	0.948598	0.457944	0.649533	5.738318	0.154054	12.83
235	114.1508	32.92335	3.142737	0.573333	38.37333	2.146667	0.986667	0.933333	5052	3.746667	0.306667	0.186667	0.413333	0.906667	0.4	0.426667	5.6	0.150838	14.43
148	119.8687	30.74474	3.14386	0.57037	34.42963	2.02716	0.977778	0.822222	9449.852	3.45679	0.059259	0.958025	0.555556	0.898765	0.224691	0.51358	5.469136	0.167281	29.86
130	118.2646	25.1928	3.145049	0.572524	35.68898	1.951883	0.889121	0.799163	7104.835	3.338215	0.020223	0.728033	0.647141	0.943515	0.230126	0.942817	5.605997	0.177042	21.32
170	119.2019	26.04819	3.149955	0.547274	34.23465	2.256729	0.701863	0.783989	7358.213	3.113182	0.023464	0.869565	0.726018	0.937888	0.332643	0.599034	5.538302	0.269861	25.03
58	113.8791	31.11831	3.150973	0.5	35.28571	2.303571	0.785714	0.839286	6235.714	3.339286	0.071429	0.303571	0.696429	0.839286	0.553571	0.714286	5.375	0.084556	11.78
199	125.3055	42.81082	3.152436	0.611111	37.88889	1.777778	1	0.833333	5805.556	3.333333	0	0.222222	0	1	0.555556	0.166667	5.611111	0.14699	15.5
50	120.7793	30.62213	3.162208	0.529412	34.60915	2.045752	0.415686	0.833987	8007.233	3.296732	0.108497	0.88366	0.419608	0.901961	0.220915	0.467974	5.594771	0.202782	34.32
141	116.7678	32.47312	3.165263	0.495238	37.46667	2.07619	0.714286	0.933333	5761.905	3.495238	0.361905	0.161905	0.580952	0.857143	0.419048	0.219048	5.504762	0.174511	17.07
230	113.4242	31.82915	3.16833	0.741379	36.91379	2.258621	0.913793	0.844828	8806.897	4.103448	0.017241	0.310345	0.448276	0.948276	0.189655	1.086207	5.862069	0.177492	14.17
201	119.1388	34.53585	3.172527	0.617021	40.09575	2.106383	1	0.840426	7051.064	3.446809	0.308511	0.43617	0.265957	0.87234	0.478723	0.648936	5.414894	0.189978	18.06
63	121.479	29.73048	3.172685	0.514804	36.44532	1.958308	0.965559	0.841088	7481.668	3.252568	0.045317	0.981873	0.410876	0.912387	0.209668	0.471903	5.492447	0.239399	40.23
97	114.5027	23.23511	3.172746	0.517327	34.39356	2.269802	0.965347	0.752475	7508.663	3.279703	0.049505	0.522277	0.767327	0.962871	0.247525	1.163366	5.992574	0.270376	29.19
25	103.638	36.35519	3.174401	0.510578	36.00071	2.37165	0.933004	0.847673	5963.581	3.270099	0.207334	0.651622	0.391396	0.836389	0.415374	0.540197	5.550071	0.236121	22.51
210	113.4727	34.6255	3.174875	0.522714	33.58584	2.6	0.978761	0.733923	6984.175	3.174631	0.221829	0.585251	0.297345	0.943953	0.356342	0.780531	5.893215	0.239414	41.72
84	107.5905	36.16249	3.175476	0.577039	35.72508	2.202417	0.978852	0.827795	5588	3.280967	0.181269	0.643505	0.274924	0.94864	0.462236	0.667674	5.800604	0.155482	11.2
120	109.566	38.03854	3.177804	0.548563	36.39083	2.009324	0.961927	0.917638	5128.6	3.451437	0.086247	0.581197	0.119658	0.961927	0.529138	0.459984	5.710956	0.184983	14.31
30	118.1421	27.33945	3.181093	0.623077	39.56923	2.053846	0.730769	0.869231	7820.769	3.430769	0.123077	0.538462	0.838462	0.946154	0.361538	0.607692	5.569231	0.198736	16.26
223	113.153	28.22712	3.181996	0.539541	36.19324	2.594388	0.970663	0.79273	7375.268	3.006378	0.158163	0.507653	0.461735	0.934311	0.418367	0.936224	5.941327	0.163818	38.52

129	116.7656	38.26868	3.189697	0.490446	35.42038	2.375796	0.968153	0.834395	6098.089	3.159236	0.388535	0.43949	0.585987	0.936306	0.566879	0.324841	5.426752	0.199072	17.85
112	119.471	29.8999	3.189795	0.52945	35.04401	2.311974	0.957929	0.784466	9400.095	3.203883	0.064725	0.961165	0.453722	0.893204	0.352751	0.473786	5.53657	0.361682	42.55
38	114.8246	26.97341	3.190271	0.54023	32.58046	2.672414	0.896552	0.729885	7873.563	3.718391	0.103448	0.229885	0.655172	0.890805	0.557471	1.155172	6.488506	0.150239	13.64
88	109.3153	36.43714	3.199989	0.507886	38.37539	1.899054	0.981073	0.933754	5154.858	3.438486	0.056782	0.337539	0.164038	0.974763	0.536278	0.37224	5.542587	0.13307	16.49
216	110.8744	26.91324	3.202049	0.536	36.68	2.448	0.984	0.808	6106.4	3.68	0.136	0.24	0.6	1	0.608	0.48	5.592	0.159654	17.25
218	109.0332	35.19603	3.205546	0.419355	39.45161	2.16129	0.967742	0.83871	4351.613	3.129032	0.129032	0.612903	0.387097	0.903226	0.451613	0.806452	5.870968	0.121016	18.55
7	117.3958	26.29946	3.206931	0.546012	38.1227	2.190184	0.245399	0.822086	6900	3.282209	0.269939	0.613497	0.840491	0.920245	0.368098	0.754601	5.760736	0.175134	16.84
178	104.6828	29.2868	3.21014	0.56	41.36	2.42	0.76	0.8	6696	3.1	0.48	0.56	0.14	0.84	0.58	0.6	5.6	0.164722	14.63
123	116.5953	23.33019	3.214452	0.542373	36.01695	1.847458	0.966102	0.898305	6486.441	4.288136	0.033898	0.491525	0.338983	0.932203	0.169492	0.559322	5.254237	0.362317	18.04
60	111.6131	27.739	3.214559	0.528796	38.6911	2.230366	0.968586	0.863874	5240.209	3.560209	0.277487	0.308901	0.418848	0.942408	0.549738	0.575916	6.04712	0.156821	14.92
207	114.8166	37.21219	3.218303	0.487805	37.08537	2.414634	0.97561	0.902439	5298.171	3.402439	0.243902	0.567073	0.579268	0.969512	0.469512	0.780488	5.5	0.166391	8.71
34	121.0444	32.18304	3.224616	0.526132	35.72822	2.003484	0.989547	0.825784	7662.892	3.355401	0.090592	0.832753	0.418118	0.912892	0.240418	0.250871	5.362369	0.171836	21.06
99	119.4744	32.73729	3.232034	0.512346	37.76543	2.123457	0.932099	0.845679	6598.333	3.104938	0.222222	0.641975	0.376543	0.87037	0.444444	0.395062	5.660494	0.210724	20.29
86	114.5351	34.60491	3.235912	0.484848	35.81818	2.378788	0.969697	0.878788	6301.515	3.80303	0.363636	0.606061	0.333333	0.969697	0.318182	0.909091	5.954545	0.17909	4.19
242	115.3398	30.71608	3.237182	0.510204	34.96429	2.678571	0.867347	0.923469	7230.102	3.709184	0.428571	0.188776	0.596939	0.693878	0.622449	0.591837	5.954082	0.137703	15.83
174	120.6366	29.73461	3.248846	0.534413	36.44939	2.018219	0.963563	0.819838	8158.219	3.362348	0.032389	0.975709	0.510121	0.919028	0.246964	0.688259	5.477733	0.165249	25.49
77	119.6365	31.62495	3.250613	0.515674	35.74608	2.133229	0.987461	0.854232	7841.066	3.357367	0.100313	0.931034	0.492163	0.924765	0.341693	0.404389	5.460815	0.179982	40.1
126	115.5329	23.00683	3.25194	0.484848	34.42424	2.333333	0.969697	0.575758	7963.636	3.939394	0.666667	0.30303	0.787879	0.878788	0.727273	0.484848	5.272727	0.253805	9.97
145	114.1372	22.64532	3.253914	0.493542	33.24405	2.67845	0.944256	0.776343	10294	3.274643	0.057784	0.633583	0.764786	0.852481	0.420802	1.046227	5.966689	0.905512	87.31
179	120.6559	31.38345	3.256705	0.518405	34.74049	2.371779	0.912883	0.853374	8750.337	3.269325	0.236196	0.930061	0.528221	0.899387	0.368712	0.757055	5.66319	0.254073	49.76
194	108.7916	34.10707	3.256996	0.530519	34.83377	2.534416	0.964286	0.824026	5760.928	3.081818	0.105844	0.732468	0.083766	0.927922	0.372727	0.588312	5.694156	0.237548	35.69
244	126.9861	49.2631	3.258187	0.5	41.12963	1.62963	1	0.851852	4779.63	2.685185	0.277778	0.388889	0.148148	0.777778	0.5	0.259259	5.462963	0.124837	10.03
32	116.0186	28.64979	3.260829	0.53405	35.82581	2.295341	0.864516	0.768459	6927.09	3.230824	0.124014	0.575627	0.658065	0.858065	0.406452	0.81147	5.796416	0.226328	28.08
215	102.0435	38.43221	3.262142	0.536842	40.14737	1.947368	0.989474	0.936842	4870.611	2.873684	0.6	0.894737	0.452632	0.926316	0.547368	0.694737	5.831579	0.110408	5.69
19	112.9445	23.00653	3.265397	0.515546	35.53001	2.14389	0.97397	0.82791	7316.22	3.419378	0.068691	0.686913	0.479393	0.912509	0.272596	0.921909	5.903832	0.234244	42.72
8	113.3936	22.51988	3.26667	0.49434	34.85472	2.177358	0.969811	0.815094	7260.66	3.526415	0.169811	0.7	0.6	0.886792	0.288679	0.654717	5.873585	0.223246	56.11
193	101.4369	36.8258	3.26771	0.540909	36.76	2.087273	0.97	0.867273	6974.842	3.463636	0.19	0.767273	0.65	0.891818	0.334545	0.815455	5.734545	0.221348	19.49
29	118.8427	31.92753	3.269203	0.583104	36.4478	2.421703	0.960165	0.802885	8586.913	2.960852	0.091346	0.875687	0.511676	0.92033	0.42239	0.611264	5.820055	0.287011	75.36
18	115.1712	39.02159	3.270692	0.544444	34.60278	2.244444	0.969444	0.719444	5878.472	2.925	0.125	0.286111	0.361111	0.930556	0.297222	0.402778	5.469444	0.217452	19.41
147	109.8531	34.95053	3.270984	0.544118	38.21078	2.421569	0.926471	0.862745	6030.392	3.573529	0.269608	0.352941	0.318627	0.931373	0.455882	0.901961	6.039216	0.126752	18.23
44	114.8776	33.72209	3.271929	0.394737	36.1579	2.394737	1	0.921053	6202.632	3.368421	0.394737	0	0.131579	0.947368	0.342105	0.263158	5.184211	0.150496	11.17
107	120.0757	31.52409	3.275646	0.549165	36.27149	2.213358	0.96475	0.861472	7877.291	3.411874	0.181818	0.934447	0.5671	0.91342	0.36611	0.533704	5.598639	0.202795	42.5
83	113.539	23.33085	3.27713	0.491525	33.15322	2.500339	0.96	0.755254	7905.344	3.104407	0.069153	0.770847	0.544407	0.88678	0.321356	0.941017	5.92678	0.318282	46.78
114	124.5684	41.22883	3.277579	0.508475	37.13559	2.169492	0.983051	0.847458	4746.949	3.033898	0.440678	0.694915	0.152542	0.949153	0.457627	0.508475	5.694915	0.137996	18.29
101	116.1198	23.3361	3.279774	0.647059	36.73529	2.176471	1	0.764706	7711.765	3.205882	0	0.705882	0.294118	0.941176	0.323529	3.029412	9.411765	0.22992	9.13
182	112.5696	29.99689	3.284361	0.513158	39.11842	1.848684	0.967105	0.940789	6673.026	3.421053	0.072368	0.388158	0.328947	0.835526	0.414474	0.388158	5.578947	0.171087	12.25
234	118.3651	31.63865	3.285062	0.529412	39.01471	1.970588	0.963235	0.963235	5921.25	3.316176	0.522059	0.514706	0.507353	0.933824	0.5	0.463235	5.588235	0.12388	18.15
46	108.3902	34.78494	3.286307	0.525822	36.15258	2.29108	0.962441	0.809859	4667.911	3.098592	0.112676	0.380282	0.140845	0.948357	0.387324	0.561033	5.821596	0.138272	13.97
131	114.9578	24.04462	3.286417	0.439394	35.5	2.106061	0.969697	0.80303	7290.909	3.621212	0.242424	0.181818	0.621212	0.909091	0.590909	0.712121	6.181818	0.211147	11.67
82	106.6482	35.30586	3.294382	0.555556	36.78571	2.234127	0.964286	0.892857	6133.929	3.361111	0.166667	0.793651	0.40873	0.960317	0.571429	0.428571	5.52381	0.135116	11.89
246	116.7389	25.29316	3.294763	0.533835	37.38346	2.112782	0.962406	0.864662	6582.707	3.503759	0.037594	0.699248	0.81203	0.984962	0.315789	0.857143	5.804511	0.218794	16.71
187	120.2049	40.61721	3.295224	0.5	39	2.222222	0.944444	0.833333	5644.444	3	0.5	0.888889	0.222222	0.777778	0.722222	0.833333	5.722222	0.174658	14.39
208	114.543	36.55246	3.300131	0.537313	39.8209	2.11194	0.970149	0.932836	4804.478	3.61194	0.149254	0.649254	0.246269	0.925373	0.171642	0.507463	5.492537	0.195595	19.7
166	120.1938	33.51557	3.301697	0.548387	34.87097	2.225806	0.935484	0.903226	10316.13	3.193548	0.096774	0.516129	0.322581	0.967742	0.322581	0.741935	5.870968	0.172731	16.23
150	118.1023	32.54419	3.304651	0.517647	36.27451	2.278431	0.952941	0.909804	5931.765	3.376471	0.486275	0.141176	0.741176	0.960784	0.682353	1.082353	5.870588	0.201035	18.06
118	113.5174	27.11881	3.307195	0.509662	38.07971	2.39372	0.963768	0.86715	7057.486	3.21256	0.31401	0.44686	0.350242	0.845411	0.495169	0.746377	5.939614	0.123509	19.75
176	122.1829	30.11824	3.31187	0.612903	36.17742	2.064516	0.967742	0.733871	7727.419	2.927419	0.048387	0.983871	0.435484	0.903226	0.387097	0.330645	5.459677	0.232996	25.21
42	111.2627	37.67871	3.312008	0.607407	36.6963	2.22963	0.911111	0.903704	5420.667	3.474074	0.02963	0.377778	0.674074	0.925926	0.496296	0.97037	5.881481	0.143052	17.69
127	112.6726	22.26739	3.313467	0.516517	35.94294	2.186186	0.993994	0.828829	6083.084	3.540541	0.225225	0.399399	0.552553	0.918919	0.363363	0.915916	5.966967	0.223599	21.62
188	117.3248	33.10936	3.316151	0.496183	37.55725	2.19084	0.992366	0.885496	6604.962	3.572519	0.541985	0.419847	0.648855	0.931298	0.572519	0.847328	5.748092	0.164602	22.64
191	112.5742	26.79434	3.317167	0.52968	38.41553	2.273973	0.958904	0.803653	5927.626	3.39726	0.26484	0.456621	0.461187	0.922374	0.621005	0.872146	6.068493	0.130255	21.21
173	126.6459	46.89376	3.320026	0.603774	42.62264	1.830189	0.226415	0.792453	3270.755	2.792453	0.150943	0.150943	0.09434	0.773585	0.679245	0.698113	5.377358	0.150192	2.25
49	118.3322	39.70915	3.321349	0.524355	38.09169	2.077364	0.95702	0.836676	5636.484	3.12894	0.140401	0.733524	0.383954	0.936963	0.389685	0.389685	5.469914	0.147192	19.88
4	111.1057	34.36115	3.3312	0.529412	39.15686	2.323529	0.970588	0.911765	4917.647	3.098039	0.166667	0.323529	0.313725	0.911765	0.529412	0.22549	5.539216	0.133588	16.04

243	115.0248	29.92757	3.333062	0.521127	39.61972	2.042254	0.816901	0.873239	6387.324	3.633803	0.098592	0.464789	0.422535	0.943662	0.521127	0.422535	5.492958	0.140679	47.96
192	111.9368	31.93231	3.33799	0.487879	36.32424	2.230303	0.981818	0.890909	5647.576	3.442424	0.133333	0.60303	0.339394	0.851515	0.518182	0.787879	5.981818	0.170711	14.28
35	112.2837	33.0443	3.338808	0.516667	40.01667	2.2875	0.945833	0.845833	7213.542	3.433333	0.145833	0.433333	0.366667	0.9375	0.5625	0.4125	5.366667	0.129532	11.27
219	121.6141	41.4626	3.340836	0.553191	41.08511	2.468085	0.893617	0.851064	6102.128	2.765957	0.446809	0.531915	0.12766	0.680851	0.638298	0.276596	5.361702	0.138334	17.12
245	124.5456	47.69965	3.342305	0.4	43.31429	2.285714	0.971429	0.942857	5594.286	2.8	0.171429	0.028571	0.114286	0.8	0.8	0.257143	5.428571	0.174802	12
96	110.0751	27.54816	3.343018	0.529148	38.04933	2.286996	0.977578	0.852018	6891.596	3.641256	0.336323	0.412556	0.565022	0.973094	0.461883	1.269058	5.730942	0.163401	15.14
233	123.0125	40.71341	3.347677	0.513514	39.36487	1.725225	0.981982	0.837838	5170.27	2.891892	0.09009	0.873874	0.085586	0.725225	0.486486	0.202703	5.198198	0.131453	10.47
108	102.8728	25.38684	3.34814	0.531559	35.39088	2.038023	0.969582	0.78327	5913.004	3.177186	0.050951	0.656274	0.263118	0.90038	0.415209	0.639544	5.726996	0.201317	28.16
185	113.9027	27.51265	3.3489	0.571429	36.63492	2.269841	0.936508	0.857143	7215.873	3.555556	0.031746	0.142857	0.507937	0.936508	0.666667	0.412698	5.603175	0.147705	17.09
202	123.2461	41.18184	3.350175	0.571429	48.91429	1.6	0.885714	0.771429	3249.143	2.542857	0.114286	0.571429	0.142857	0.742857	0.657143	0.114286	5.057143	0.144513	19.29
10	111.3767	36.22673	3.353128	0.582278	36.5865	2.232068	0.936709	0.869198	4486.709	3.481013	0.113924	0.400844	0.518987	0.932489	0.421941	0.759494	5.805907	0.145984	6.2
128	117.3642	30.28326	3.353333	0.5	33	2.44	0.92	0.92	7472	3.26	0.42	0.44	0.38	0.98	0.44	0.6	5.84	0.174192	17.05
81	113.011	33.79592	3.356479	0.530675	38.88037	2.294479	0.98773	0.855828	4436.503	3.101227	0.196319	0.435583	0.40184	0.953988	0.484663	0.361963	5.5	0.142481	12.39
161	123.0118	45.34652	3.357072	0.5	41.36719	1.929688	0.90625	0.765625	4162.32	2.65625	0.328125	0.46875	0.132813	0.851563	0.546875	0.28125	5.507813	0.16286	14.31
3	121.4842	31.21193	3.36076	0.507527	36.54648	2.38399	0.879092	0.860932	10037.36	3.270968	0.169892	0.737395	0.58638	0.876941	0.313262	0.527121	5.706571	0.38028	55.61
190	118.6745	28.93437	3.364161	0.587302	38.61905	2.507937	0	0.84127	10573.38	3.174603	0.603175	0.603175	0.920635	0.936508	0.619048	0.634921	6.253968	0.242641	20.3
47	115.6974	34.28935	3.366685	0.526882	32.65591	2.27957	0.978495	0.72043	5468.065	2.903226	0.16129	0.344086	0.268817	0.903226	0.537634	0.924731	6.021505	0.185285	13.53
197	106.7069	26.84252	3.366881	0.530839	36.84893	2.097713	0.891199	0.776854	6417.31	3.516286	0.153846	0.626473	0.241857	0.899515	0.390159	0.512128	5.70894	0.225841	33.88
103	101.7294	26.80727	3.368787	0.566775	40.86319	1.990228	0.983713	0.794788	4863.029	3.211726	0.237785	0.762215	0.540717	0.892508	0.514658	0.589577	5.557003	0.125827	18.96
45	127.9576	45.63734	3.370852	0.543672	39.39037	2.130125	0.969697	0.754011	5703.576	2.519608	0.233512	0.493761	0.072193	0.822638	0.572193	0.400178	5.418004	0.234597	23.67
115	124.4768	44.8013	3.37319	0.496032	37.06349	2	0.988095	0.81746	4325.794	3.003968	0.09127	0.5	0.007937	0.896825	0.615079	0.321429	5.392857	0.149725	12.02
186	122.4512	40.38895	3.374682	0.509804	41.17647	1.95098	0.901961	0.735294	4635.098	2.686275	0.264706	0.598039	0.186275	0.843137	0.490196	0.392157	5.313725	0.118074	20.84
240	118.0709	29.90653	3.374785	0.455621	36.82249	2.159763	0.95858	0.87574	6606.509	3.343195	0.508876	0.260355	0.686391	0.970414	0.473373	0.810651	6.076923	0.182538	21.8
102	114.0895	35.26384	3.375512	0.530864	33.02963	2.508642	0.977778	0.634568	4612.938	2.809877	0.239506	0.54321	0.311111	0.91358	0.409877	0.812346	6.049383	0.144716	5.7
12	119.512	28.19928	3.375939	0.529412	35.90498	2.226244	0.936652	0.746606	8331.222	3.289593	0.126697	0.859729	0.778281	0.954751	0.307692	0.651584	5.79638	0.235588	20.97
57	117.3416	39.28378	3.37869	0.513002	36.59128	2.195955	0.951931	0.885211	6762.413	3.291831	0.212766	0.75151	0.575256	0.861045	0.355923	0.572892	5.69845	0.38063	38.59
48	109.9009	33.64795	3.378886	0.5	39.67857	2.214286	0.964286	0.821429	5189.286	3.571429	0.071429	0.571429	0.071429	0.964286	0.428571	0.25	5.535714	0.148215	12.23
165	123.1377	42.09432	3.380262	0.50212	37.25445	2.366412	0.957591	0.841391	6084.521	2.806616	0.38592	0.610687	0.187447	0.821883	0.45123	0.356234	5.41391	0.194927	26.64
95	112.4087	38.88214	3.383459	0.547619	38.38095	2.035714	0.934524	0.904762	3974.405	3.363095	0.059524	0.452381	0.511905	0.928571	0.613095	0.666667	5.547619	0.132874	6.39
204	105.4717	30.63174	3.386475	0.465116	39.40698	2.313953	0.94186	0.872093	5365.698	3.139535	0.406977	0.244186	0.313953	0.94186	0.627907	0.639535	6.151163	0.159649	12.28
43	114.1753	29.62404	3.386981	0.526316	36.47368	2.052632	1	0.947368	8900	3.736842	0.421053	0.315789	0.684211	0.736842	0.578947	0.210526	5.526316	0.12095	19.29
222	125.7674	44.38372	3.388106	0.521671	38.56974	2.249015	0.958235	0.72892	5369.377	2.603625	0.244287	0.621749	0.226162	0.827423	0.555556	0.49409	5.567376	0.241599	23.75
132	117.0249	36.00176	3.38827	0.485714	42.48571	2.171429	0.085714	0.971429	5862.857	3.885714	0.2	0.428571	0.228571	1	0.571429	0.342857	5.714286	0.172913	18.7
66	105.9493	25.99553	3.395529	0.548148	38.13333	1.837037	0.948148	0.881481	5639.259	3.933333	0.081481	0.414815	0.237037	0.874074	0.303704	0.466667	5.8	0.187003	15.74
14	100.524	26.94065	3.400197	0.512	36.576	2.216	0.944	0.84	5271.2	3.248	0.168	0.728	0.256	0.92	0.248	0.696	5.528	0.271223	4.29
113	112.6416	39.61686	3.401319	0.49697	40.88485	2.054545	0.90303	0.878788	3925.333	3.351515	0.072727	0.260606	0.557576	0.872727	0.557576	0.484848	5.484848	0.101809	13.41
110	112.7065	35.61051	3.403121	0.503876	35.45736	2.472868	0.883721	0.872093	4289.399	3.418605	0.027132	0.817829	0.709302	0.926357	0.445736	0.899225	5.705426	0.155098	16.69
76	113.2543	29.06636	3.40451	0.586387	38.75916	2.424084	0.167539	0.900524	6810.209	3.209424	0.089005	0.167539	0.534031	0.91623	0.518325	0.942408	6.052356	0.165848	19.36
52	124.6998	46.34703	3.404892	0.495192	41.0649	1.985577	0.353365	0.858173	4294.522	2.622596	0.199519	0.838942	0.125	0.848558	0.649038	0.384615	5.454327	0.138657	16.76
136	112.0311	34.29189	3.405193	0.555324	34.08977	2.549061	0.968685	0.676409	4983.716	2.885177	0.139875	0.39666	0.336117	0.891441	0.36952	0.463466	5.60334	0.15493	19.7
198	115.273	25.70886	3.40799	0.541114	34.5756	2.286472	0.944297	0.793103	7264.085	3.416446	0.135279	0.259947	0.450928	0.944297	0.501326	1.175066	5.933687	0.209995	15.22
39	117.3549	31.76278	3.409555	0.518932	33.75729	2.563004	0.934202	0.963998	8543.987	3.274364	0.479205	0.509621	0.544382	0.96648	0.490999	0.911856	5.993793	0.28313	28.23
98	103.9296	30.65361	3.410336	0.522339	37.27595	2.358739	0.96452	0.792378	6660.545	3.07293	0.177398	0.522339	0.513141	0.905388	0.555191	0.788436	6.033509	0.224397	32.43
138	110.4187	19.85392	3.410511	0.565141	35.72183	2.547535	0.908451	0.84331	7449.451	3.237676	0.181338	0.727113	0.626761	0.823944	0.545775	1.042254	5.72007	0.359784	33.15
167	103.7521	29.92691	3.410694	0.479452	38.57534	2.287671	0.90411	0.780822	6746.575	3.178082	0.534247	0.30137	0.726027	0.917808	0.726027	0.452055	5.424658	0.184194	14.57
23	116.2288	31.65885	3.412875	0.517391	33.64783	2.626087	0.9	0.891304	7495.696	3.208696	0.669565	0.091304	0.791304	0.96087	0.465217	0.878261	6.556522	0.213834	15.65
164	104.6216	36.6135	3.416166	0.481675	38.75393	2.13089	0.963351	0.937173	5106.283	3.675393	0.481675	0.685864	0.303665	0.926702	0.513089	0.528796	5.895288	0.166323	11.56
121	114.3429	30.62405	3.416333	0.479911	35.80878	2.421875	0.947173	0.924107	8243.533	3.411458	0.252976	0.74256	0.421875	0.854911	0.456845	0.755952	5.850446	0.270568	38.33
104	114.8522	27.84532	3.418343	0.526316	37.05263	2.263158	0.982456	0.929825	6343.86	3.666667	0.368421	0.596491	0.77193	0.947368	0.894737	0.666667	6.157895	0.114709	18.97
160	113.3623	22.1519	3.41862	0.505976	35.18725	2.410359	0.940239	0.900398	8098.606	3.505976	0.091633	0.800797	0.733068	0.880478	0.398406	1.035857	6.039841	0.45816	53.03
80	106.6634	30.42638	3.419021	0.473684	39.10526	2.736842	0.842105	0.842105	7173.684	3.052632	0.315789	0.368421	0.684211	1	0.947368	1.578947	6.578947	0.137541	13.65
134	117.2155	36.63672	3.419228	0.467692	34.61785	2.709538	0.070154	0.900923	7343.6	3.030769	0.249846	0.929846	0.416	0.902769	0.478769	0.756923	5.936	0.206766	44.06
94	107.0375	32.01717	3.425124	0.566038	40.18868	2.283019	0.981132	0.867925	6470.755	3.716981	0.622642	0.075472	0.735849	0.962264	0.528302	0.54717	6.056604	0.143146	12.85
61	119.4905	26.97081	3.425601	0.506173	36.14815	2.08642	0.703704	0.839506	8613.58	3.506173	0.17284	0.320988	0.740741	0.938272	0.320988	1.037037	5.814815	0.235493	16.23

146	112.6029	27.73161	3.42628	0.556338	35.85916	2.704225	0.992958	0.830986	8062.676	3.105634	0.147887	0.359155	0.507042	0.964789	0.492958	0.957746	6.070423	0.140339	19.75
15	103.5695	29.21584	3.426283	0.5	38.14815	2.425926	0.842593	0.777778	5186.111	2.898148	0.416667	0.296296	0.666667	0.787037	0.564815	0.425926	5.731481	0.1589	14.3
168	121.9942	41.06603	3.428255	0.508772	41.01754	1.982456	0.929825	0.894737	5109.474	2.877193	0.666667	0.684211	0.368421	0.561404	0.45614	0.526316	5.473684	0.108513	22.69
177	118.1348	31.16237	3.431629	0.505133	34.8809	2.447639	0.942505	0.880903	7215.084	3.37577	0.37577	0.49076	0.652977	0.936345	0.519507	0.673511	5.973306	0.193234	19.02
209	113.1363	25.81447	3.433547	0.542017	38.68908	2.319328	0.987395	0.806723	6809.958	3.584034	0.226891	0.357143	0.445378	0.941176	0.5	1.159664	6.054622	0.142808	23.86
236	132.2859	45.62898	3.439523	0.396226	40.07547	2.056604	0.981132	0.716981	3790.679	2.584906	0.528302	0.358491	0.188679	0.867925	0.641509	0.226415	5.509434	0.162029	6.1
162	111.9514	28.57521	3.440092	0.393443	33.96721	2.606557	0.983607	0.819672	7747.541	3.737705	0.229508	0.131148	0.606557	1	0.737705	0.852459	5.918033	0.148254	13.21
27	116.4126	40.18561	3.440396	0.525991	36.34512	2.545929	0.917399	0.832186	9106.9	2.956563	0.084026	0.816758	0.462141	0.913601	0.388797	0.654166	5.854261	0.515015	57.75
180	110.9535	22.01009	3.440871	0.592593	35	3.148148	0.740741	0.666667	5985.185	2.703704	0.111111	0.444444	0.703704	0.740741	0.62963	0.888889	7.333333	0.242777	11.42
59	112.3155	37.95912	3.441725	0.50392	35.53172	2.535994	0.920884	0.884533	5673.77	3.143977	0.184604	0.74412	0.388453	0.898788	0.424804	0.760513	5.634355	0.286533	28.52
64	108.9262	32.75748	3.442038	0.653846	41.11539	1.923077	0.923077	0.653846	4453.846	2.307692	0.192308	0.653846	0.269231	0.884615	0.192308	0.884615	6	0.168422	12.07
157	113.1304	35.11157	3.442531	0.522059	37.88235	2.154412	0.985294	0.852941	4677.206	3.477941	0.198529	0.610294	0.580882	0.830882	0.360294	0.404412	5.647059	0.154999	15.74
122	103.0572	38.17552	3.444444	0.527778	38.91667	1.777778	1	0.944444	4522.222	4.138889	0.194444	0.972222	0.25	0.944444	0.388889	2.388889	7.833333	0.203351	10.45
171	115.8818	36.45863	3.445784	0.513889	37.20833	2.416667	0.125	0.875	7809.722	3.763889	0.736111	0.569444	0.083333	0.930556	0.486111	0.902778	5.847222	0.191392	24.8
67	114.3536	35.87704	3.448574	0.496183	39.0229	2.022901	0.992366	0.908397	4773.282	3.610687	0.251908	0.748092	0.351145	0.938931	0.251908	0.580153	5.824427	0.130568	22.9
41	126.8433	43.57963	3.452385	0.496	39.076	2.312	0.956	0.764	4962.12	2.792	0.548	0.544	0.116	0.856	0.672	0.444	5.516	0.184591	6.68
151	117.8412	37.53944	3.460671	0.475728	31.31068	2.572816	0.23301	0.805825	6813.592	2.941748	0.553398	0.38835	0.679612	0.902913	0.427184	0.883495	6.524272	0.138522	14.84
73	118.8517	30.68571	3.461747	0.518987	36.12658	2.512658	0.905063	0.955696	8886.076	3.405063	0.367089	0.411392	0.651899	0.936709	0.664557	0.898734	6.202532	0.155182	19.36
72	107.2008	34.37894	3.462907	0.553571	32.45536	2.366071	0.991071	0.660714	4374.107	2.758929	0.455357	0.410714	0.142857	0.982143	0.5	0.732143	5.883929	0.13227	5.04
124	107.103	33.09052	3.473183	0.5	38.23469	2.306122	0.969388	0.816327	5912.918	3.05102	0.163265	0.387755	0.132653	0.969388	0.520408	0.795918	6.102041	0.129311	14.82
13	118.6168	37.63654	3.473788	0.5	34.83088	2.625	0.022059	0.889706	6700	3.323529	0.566176	0.772059	0.5	0.970588	0.544118	0.897059	5.992647	0.12135	24.2
109	112.9621	37.32672	3.475075	0.508621	38.26293	2.258621	0.87931	0.922414	4189.655	3.219828	0.211207	0.49569	0.487069	0.892241	0.646552	0.922414	5.650862	0.143985	19.01
31	110.4415	32.45681	3.476462	0.47695	36.30851	2.276596	0.95922	0.874113	5481.454	3.375887	0.37766	0.430851	0.661348	0.851064	0.521277	0.843972	5.900709	0.161302	15.2
220	117.5556	30.88462	3.479814	0.482759	36.2069	2.310345	0.87931	0.95977	6273.851	3.310345	0.517241	0.431034	0.752874	0.942529	0.58046	0.701149	5.896552	0.154854	18.36
2	109.4177	18.36384	3.47984	0.578261	35.97174	2.445652	0.95	0.91087	9087.5	3.513043	0.130435	0.517391	0.46087	0.873913	0.406522	0.667391	5.841304	0.766811	25.85
163	127.2853	42.06961	3.498754	0.548387	42.14516	1.629032	0.596774	0.83871	3962.903	2.967742	0.5	0.483871	0.16129	0.709677	0.387097	0.564516	5.483871	0.137999	15.7
149	110.1649	21.07535	3.50421	0.485714	36.28571	2.228571	0.971429	0.971429	5314.286	4.285714	0.2	0.142857	0.914286	0.942857	0.514286	0.514286	5.914286	0.266489	10.14
211	114.7542	30.32898	3.505375	0.548387	40.67742	2.064516	1	0.903226	5348.387	3.580645	0.225806	0.709677	0.645161	0.967742	0.193548	0.451613	5.580645	0.204939	16.59
33	106.2037	31.19573	3.506782	0.519231	39.71154	2.153846	0.980769	0.846154	5790.385	3.25	0.326923	0.057692	0.326923	0.865385	0.615385	0.903846	5.923077	0.168496	12.69
36	118.1203	24.66184	3.507014	0.523749	35.35909	2.358455	0.981001	0.858138	9100.939	3.349588	0.083597	0.915769	0.852438	0.960101	0.340722	1.252692	5.962635	0.451808	55.84
217	124.1707	42.64388	3.507936	0.571429	43.80952	2.047619	1	0.809524	4414.286	2.380952	0.761905	0.380952	0.238095	0.952381	0.52381	0.190476	5.714286	0.167459	16.62
224	112.9158	36.47599	3.508487	0.559783	36.75544	2.179348	0.945652	0.869565	5441.554	3.565217	0.059783	0.836957	0.342391	0.961957	0.353261	1.043478	5.581522	0.134263	18.04
137	116.736	35.36895	3.509617	0.509259	36.33333	2.37963	0.055556	0.962963	5823.148	3.527778	0.305556	0.574074	0.740741	0.972222	0.425926	0.712963	5.962963	0.145312	17.25
212	107.8745	30.05716	3.510384	0.494806	37.82042	2.421193	0.936183	0.768477	6537.785	3.166518	0.450876	0.437222	0.871475	0.928169	0.638765	0.623034	5.964084	0.210976	19.97
125	111.7294	25.77332	3.523544	0.533333	36.71333	2.393333	0.973333	0.8	6588.667	3.6	0.306667	0.4	0.78	0.96	0.426667	0.786667	6	0.144672	13.91
53	113.7193	39.90043	3.525026	0.513552	42.97004	1.868759	0.96291	0.89729	3790.869	3.136947	0.126961	0.480742	0.487874	0.917261	0.67475	0.450785	5.462197	0.172227	13.88
153	113.9	33.65562	3.525301	0.473684	28.86842	2.368421	1	0.657895	4813.158	2.578947	0	0.210526	0.5	1	0.5	0.868421	6.789474	0.152155	19.84
93	104.4348	31.13005	3.529266	0.534884	38.97674	2.503876	0.945736	0.782946	5049.612	2.806202	0.387597	0.325581	0.418605	0.899225	0.697674	0.534884	5.906977	0.130597	18.49
119	116.0794	24.20307	3.529411	0.352941	40.29412	2	1	0.882353	9000	3.470588	0.352941	0.117647	0.941176	1	1	1.411765	5.176471	0.223432	11.03
65	116.486	30.57553	3.532393	0.53012	37.46386	2.349398	0.951807	0.921687	8110.482	3.626506	0.512048	0.39759	0.481928	0.927711	0.584337	0.753012	5.89759	0.19184	15.65
79	105.7823	32.26219	3.535938	0.575758	35.66667	2.363636	1	0.787879	6478.788	3.393939	0.181818	0.272727	0.545455	0.969697	0.606061	1.454545	6	0.156226	17.11
142	118.9649	33.35176	3.539203	0.461538	34.92308	2.115385	1	0.961538	6530.769	3.653846	0.307692	0.653846	0.5	0.846154	0.346154	0.423077	5.692308	0.182011	13.98
75	117.2092	33.86025	3.54023	0.551724	36.72414	2.413793	0.965517	0.931034	5544.828	3.310345	0.206897	0.62069	0.724138	1	0.896552	1.206897	6.310345	0.178573	26.28
55	122.1914	39.5848	3.540925	0.51034	39.22747	2.138848	0.934269	0.769572	5742.648	2.661743	0.337518	0.692762	0.388479	0.864845	0.45938	0.488183	5.59675	0.254145	26.61
62	121.9957	37.11747	3.545786	0.537572	35.72254	2.190751	0.32948	0.895954	6350.289	3.150289	0.416185	0.589595	0.421965	0.780347	0.369942	0.473988	5.936416	0.127228	24.57
227	115.7041	32.91698	3.54588	0.52069	35.61724	2.296552	0.975862	0.917241	6548.966	3.562069	0.358621	0.210345	0.586207	0.965517	0.603448	0.648276	5.851724	0.239924	14.78
172	104.7029	31.84913	3.548522	0.488584	38.35616	2.456621	0.936073	0.785388	5699.041	3.059361	0.310502	0.561644	0.447489	0.90411	0.671233	0.780822	6.114155	0.158381	15.46
78	111.5196	29.29995	3.54881	0.441341	37.27933	2.564246	0.972067	0.826816	7441.62	3.385475	0.195531	0.301676	0.486034	0.899441	0.52514	0.98324	6.100559	0.174246	10.19
156	115.2816	35.8088	3.549022	0.434426	34.52869	2.331967	0.991803	0.786885	5473.77	3.184426	0.286885	0.360656	0.254098	0.95082	0.364754	0.864754	6.090164	0.159107	11.94
92	116.6474	37.2486	3.549288	0.471014	33.77536	2.623188	0.144928	0.963768	6292.029	3.5	0.898551	0.304348	0.369565	0.92029	0.391304	0.695652	6.094203	0.18681	23.14
117	117.393	34.91618	3.549631	0.516129	36.41936	2.225806	0.129032	0.903226	4838.71	3.354839	0.516129	0.806452	0.548387	0.935484	0.258065	0.806452	5.548387	0.152687	20.27
232	102.6627	29.91316	3.555279	0.528302	36.73585	2.528302	0.886792	0.754717	5252.83	2.867925	0.150943	0.301887	0.716981	0.867925	0.867925	0.415094	6.358491	0.172712	17.13
231	120.1462	36.44717	3.559272	0.493151	34.63014	2.487195	0.811197	0.90947	7734.783	3.209053	0.222752	0.833234	0.52293	0.934485	0.503276	0.656939	5.849911	0.222952	33.32
203	111.0676	35.1873	3.56065	0.507813	33.67969	2.585938	0.992188	0.726563	5716.406	2.984375	0.0625	0.59375	0.320313	0.921875	0.375	1.484375	5.8125	0.123249	4.22

26	104.9111	29.64071	3.563229	0.465753	41.12329	2.232877	0.931507	0.821918	5658.219	3.082192	0.383562	0.315068	0.39726	0.890411	0.69863	0.767123	6.013699	0.144574	14.81
91	117.5177	34.35528	3.567731	0.551181	33.91339	2.771654	0.944882	0.787402	7015.748	2.96063	0.220472	0.409449	0.401575	0.944882	0.472441	0.519685	5.913386	0.202278	24.38
24	104.8913	26.1361	3.573734	0.538824	36.44706	2.004706	0.936471	0.818824	4908.042	3.955294	0.115294	0.456471	0.414118	0.938824	0.411765	0.762353	5.828235	0.131468	12.68
205	119.1874	40.08524	3.577056	0.527473	40.69231	2.274725	0.923077	0.879121	5389.011	3.032967	0.549451	0.769231	0.516484	0.945055	0.505495	0.296703	5.582418	0.20064	20.95
228	113.4994	38.06317	3.579049	0.546763	40.09353	2.18705	0.856115	0.928058	3567.77	3.244604	0.107914	0.482014	0.503597	0.856115	0.597122	0.618705	5.57554	0.138537	20.53
196	105.1155	30.10048	3.58659	0.474576	44.23729	2.101695	0.983051	0.881356	4870.763	3.644068	0.423729	0.254237	0.59322	0.932203	0.728814	0.644068	5.79661	0.143195	11.21
238	130.8005	47.63981	3.589373	0.485714	46.4	1.514286	1	0.942857	2718	2.542857	0.714286	0.114286	0	0.885714	0.6	0.428571	5.314286	0.142162	5.93
22	114.8835	32.08133	3.59152	0.555556	32.61111	2.25	1	0.75	6682.389	3.5	0.305556	0.25	0.361111	0.972222	0.5	0.75	6.222222	0.155868	5.15
71	111.1355	30.74765	3.592617	0.484163	39.61312	2.196833	0.966063	0.918552	6210.204	3.319005	0.253394	0.717195	0.337104	0.780543	0.477376	0.739819	5.970588	0.177528	17.29
206	107.0853	28.16879	3.596296	0.47151	36.86752	2.118234	0.945869	0.830484	6898.202	3.806268	0.344729	0.361823	0.321937	0.91453	0.575499	0.707977	5.789174	0.135496	18.65
69	104.6352	28.57371	3.607098	0.475845	37.99759	2.031401	0.961353	0.818841	5306.546	3.217391	0.251208	0.60628	0.73913	0.971014	0.671498	0.710145	5.789855	0.162917	14.42
183	112.6087	31.07062	3.607148	0.530864	37.48148	2.345679	0.925926	0.938272	7149.383	3.358025	0.641975	0.259259	0.802469	0.679012	0.716049	1.716049	6.296296	0.130536	16.28
51	124.4677	43.57059	3.616361	0.565217	37.45652	2.413043	0.869565	0.826087	5567.391	2.913043	0.282609	0.630435	0.195652	0.847826	0.456522	0.369565	5.695652	0.140542	7.45
237	113.7745	24.82037	3.621253	0.514286	35.8	2.085714	0.971429	0.914286	6360	3.771429	0	0.485714	0.657143	0.914286	0.457143	1.171429	7.2	0.185226	21.92
116	120.0144	41.51086	3.626679	0.586207	36.58621	2.482759	0.793103	0.931034	5168.966	3.517241	1	0.034483	0.206897	0.965517	0.448276	0.862069	5.172414	0.141353	15.38
159	120.7998	37.24463	3.63602	0.494709	34.86773	2.518519	0.113757	0.902116	6404.233	3.079365	0.412698	0.595238	0.478836	0.888889	0.436508	0.730159	5.756614	0.152019	19.36
195	113.7097	34.07235	3.64763	0.402985	34.79105	2.179104	0.970149	0.80597	5067.164	2.447761	0.328358	0.492537	0.38806	0.895522	0.238806	0.895522	6.402985	0.161626	14.99
54	98.21052	39.82837	3.651786	0.487437	35.60804	2.231156	0.98995	0.964824	6343.719	3.512563	0.673367	0.81407	0.125628	0.693467	0.301508	0.708543	6.015075	0.09468	17.04
40	132.6007	46.71905	3.668209	0.6	43.2	1.64	0.84	0.96	4004	3.04	0.16	0.36	0.08	0.68	0.44	0.2	5.52	0.108628	10.04
9	118.2806	35.30939	3.695991	0.494186	35.57558	2.593023	0.046512	0.854651	7033.721	3.122093	0.244186	0.360465	0.412791	0.930233	0.604651	0.767442	6.075581	0.143299	13.92
139	116.7433	33.72845	3.701349	0.512821	35.35897	2.205128	1	0.769231	4856.41	3.589744	0.641026	0.589744	0.487179	0.846154	0.589744	1.74359	7.153846	0.181114	14.49
154	119.0726	36.55152	3.712168	0.47093	34.80814	2.627907	0.052326	0.94186	6733.14	3.296512	0.80814	0.459302	0.540698	0.825581	0.744186	0.877907	5.837209	0.132393	21.87
106	119.1411	35.57811	3.718341	0.515152	35.09091	2.878788	0.121212	1	9148.485	3.636364	0.727273	0.666667	0.363636	0.454545	0.666667	0.909091	5.939394	0.185287	5.98
200	107.6448	31.3663	3.725664	0.530973	41.28319	2.044248	0.99115	0.761062	5186.77	3.176991	0.415929	0.256637	0.380531	0.884956	0.530973	0.787611	5.628319	0.17482	10.37
213	96.05605	40.26036	3.730444	0.561151	37.8705	2.143885	1	0.848921	5593.525	3.928058	0.489209	0.611511	0.215827	0.877698	0.776978	0.661871	5.719424	0.115833	11.76
20	132.1868	47.23527	3.743798	0.533333	43.73333	1.733333	0.966667	0.8	3082.667	2.7	0.233333	0.366667	0	0.866667	0.933333	0.066667	5.1	0.152058	14.76
184	115.6925	35.15134	3.759796	0.527778	35.80556	2.638889	0	0.972222	6107.389	3.694444	0.638889	0.611111	0.25	0.805556	0.611111	0.722222	6.25	0.15724	13.63
158	129.847	44.5882	3.761403	0.5	45.51852	1.666667	0.981481	0.814815	3681.111	2.62963	0.37037	0.333333	0	0.981481	0.648148	0.388889	5.425926	0.127711	7.17
135	105.6662	28.43989	3.767402	0.459459	38.64865	2.324324	0.932432	0.851351	5858.108	3.5	0.256757	0.324324	0.72973	0.972973	0.540541	0.864865	6.621622	0.151545	13.82
140	118.0533	36.60786	3.782368	0.494949	36.30303	2.151515	0.060606	0.979798	6671.717	3.373737	0.232323	0.858586	0.686869	0.939394	0.535354	0.767677	5.909091	0.156128	26.19
28	129.2124	47.81302	3.801287	0.533333	45.16667	1.433333	0.933333	0.666667	1756.667	3.1	0.033333	0.766667	0.033333	0.8	0.8	0.233333	5.466667	0.147618	13.97
21	116.1802	33.43549	3.827113	0.454545	33.09091	2.393939	0.787879	0.909091	6872.727	3.424242	0.272727	0.545455	0.484848	0.969697	0.515152	0.272727	5.69697	0.212618	12.93
